# Finding Key Factors for Efficient Water and Methanol Activation at Metals, Oxides, MXenes, and Metal/Oxide Interfaces

**DOI:** 10.1021/acscatal.1c03405

**Published:** 2022-01-05

**Authors:** Hai-Yan Su, Keju Sun, Xiang-Kui Gu, Sha-Sha Wang, Jing Zhu, Wei-Xue Li, Chenghua Sun, Federico Calle-Vallejo

**Affiliations:** † School of Chemical Engineering and Energy Technology, Dongguan University of Technology, Dongguan 523808, China; ‡ Key Laboratory of Applied Chemistry, College of Environmental and Chemical Engineering, Yanshan University, 438 Hebei Avenue, Qinhuangdao 066004, China; § Department of Chemical Physics, College of Chemistry and Materials Science, Hefei National Laboratory for Physical Sciences at the Microscale, iChEM, CAS Center for Excellence in Nanoscience, University of Science and Technology of China, Hefei 230026, China; ∥ Centre for Translational Atomaterials, Swinburne University of Technology, Hawthorn, VIC 3122, Australia; ⊥ Department of Materials Science and Chemical Physics & Institute of Theoretical and Computational Chemistry (IQTCUB), University of Barcelona, Martí i Franquès 1, 08028 Barcelona, Spain

**Keywords:** H_2_O dissociation, CH_3_OH dissociation, oxides, metal/oxide interfaces, proton-like hydrogen transfer, O−H bond rotation, DFT calculations

## Abstract

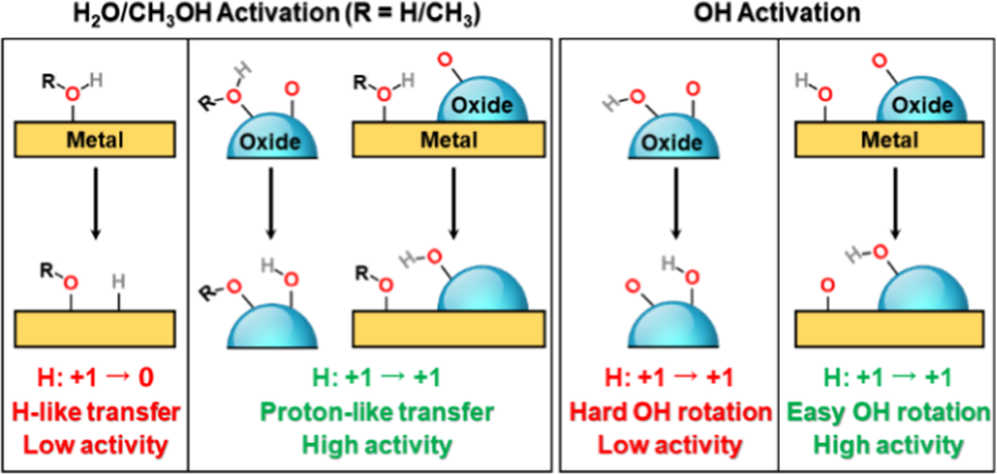

Activating water and methanol is crucial in numerous catalytic, electrocatalytic, and photocatalytic reactions. Despite extensive research, the optimal active sites for water/methanol activation are yet to be unequivocally elucidated. Here, we combine transition-state searches and electronic charge analyses on various structurally different materials to identify two features of favorable O–H bond cleavage in H_2_O, CH_3_OH, and hydroxyl: (1) low barriers appear when the charge of H moieties remains approximately constant during the dissociation process, as observed on metal oxides, MXenes, and metal/oxide interfaces. Such favorable kinetics is closely related to adsorbate/substrate hydrogen bonding and is enhanced by nearly linear O–H–O angles and short O–H distances. (2) Fast dissociation is observed when the rotation of O–H bonds is facile, which is favored by weak adsorbate binding and effective orbital overlap. Interestingly, we find that the two features are energetically proportional. Finally, we find conspicuous differences between H_2_O/CH_3_OH and OH activation, which hints toward the use of carefully engineered interfaces.

## 1 Introduction

Water plays a crucial role in numerous catalytic reactions. It can either act as a reactant for surface reactions such as the water–gas shift and methane/methanol steam reforming or facilitate reactions as moisture in the reactant gases.^1−8^ In addition, it is used as a solvent in countless inorganic and organic reactions and is also important in electrochemistry, fuel cells, and corrosion science and technology.^9,10^ Furthermore, apart from being a commodity chemical, methanol has attracted great interest in recent years for hydrogen production via methanol steam reforming, the development of direct methanol fuel cells to be used in small portable devices, and the potential of CH_3_OH photocatalytic oxidation.^2,11−15^ In view of their high thermodynamic stability, the activation of water or methanol is habitually a decisive part of catalytic pathways, often the rate-limiting step.^1,2,13,16−20^

Although numerous studies have been devoted to identifying the active sites for water and methanol activation, they are still a matter of debate in view of the coexistence of numerous structural motifs at catalytic surfaces. For instance, some authors have suggested that oxide supports (e.g., TiO_2–*x*_, CeO_2–*x*_) are responsible for water activation in the water–gas shift.^16,21−23^ Others have shown that both metals and oxide supports at metal/oxide interfaces (e.g., Cu/FeO*_x_*, Ni/TiO_2–*x*_, Au/TiO_2–*x*_) directly participate in water activation.^24−26^ Moreover, some authors claim that metals (Cu) or metal cations (Pt^δ+^, Au^δ+^) are the active sites for the water–gas shift.^27−29^ In addition, discrepancies exist about methanol activation on CuZn alloy sites or Cu/ZnO interfaces at Cu/ZnO catalysts during catalytic methanol steam reforming.^30−35^ These conflicting views greatly hamper the design and implementation of improved catalysts and call for fundamental studies that outline the different interactions between H_2_O/CH_3_OH and various structural motifs/sites. In this context, the challenge lies in identifying the common features of swift activation kinetics among structurally different materials.

In this study, we identify two such features among metals, oxides, MXenes, and metal/oxide interfaces combining the climbing-image nudged elastic band (CI-NEB) method^36^ for the location of transition states (TSs) and the Bader charge analysis.^37^ Specifically, Cu(111), Co(0001), Pt(111), rutile TiO_2_(110), and Ti_3_C_2_O_2_(0001), together with Pt/FeO and Cu/ZnO interfaces are used to model various structural motifs/sites in view of their superior performance in relevant applications, such as water–gas shift, methanol steam reforming, and CH_3_OH photocatalytic oxidation.^1,2,15^

The first feature refers to the charge state of H moieties, as there are clear energetic differences between H-like transfer and proton-like transfer during H_2_O, OH, and CH_3_OH activation. O–H bond scission in H_2_O and CH_3_OH on metals occurs via a H-like transfer process with large associated energy barriers. Conversely, such cleavage on oxides, MXenes, and metal/oxide interfaces occurs via a proton-like transfer with small barriers. The second feature refers to O–H bond rotation. Weak binding and effective orbital overlap between O atoms in OH and substrates are found to facilitate O–H bond rotation and dissociation. Finally, we show that although H_2_O, CH_3_OH, and OH interactions with metals, oxides, MXenes, and metal/oxide interfaces are fundamentally different, the two aforementioned features are energetically proportional.

## 2 Methods

Spin-unrestricted density functional theory (DFT) calculations were performed with the Vienna Ab initio Simulation Package (VASP).^38^ The interaction between ionic cores and valence electrons was described by the projector-augmented wave (PAW) method,^39^ and the Kohn–Sham valence electronic wavefunction was expanded using a plane-wave basis set with a kinetic energy cutoff of 400 eV. Exchange–correlation effects on the total energies were calculated within the generalized gradient approximation (GGA) using the Perdew–Burke–Ernzerhof (PBE) exchange–correlation functionals.^40^Section S9 in the Supporting Information (SI) shows that the effect of D3 dispersion corrections on the adsorption energies is mostly a constant downward shift with a minor effect on the trends.^41^ The total energies were converged to within 10^–4^ eV, and the forces on the atoms were converged to within 0.05 eV/Å. The lattice constants for bulk Cu (face-centered cubic (fcc)), Co (hexagonal close-packed (hcp)), Pt (fcc), TiO_2_ (rutile), and the Ti_3_C_2_O_2_ (MXene) were calculated to be 3.64, 2.50/4.03, 3.99, 4.67/2.97, and 3.04 Å, in line with the experimental values of 3.62, 2.51/4.06, 3.92, 4.59/2.96, and 3.057 Å.^42,43^

**Figure 1:**
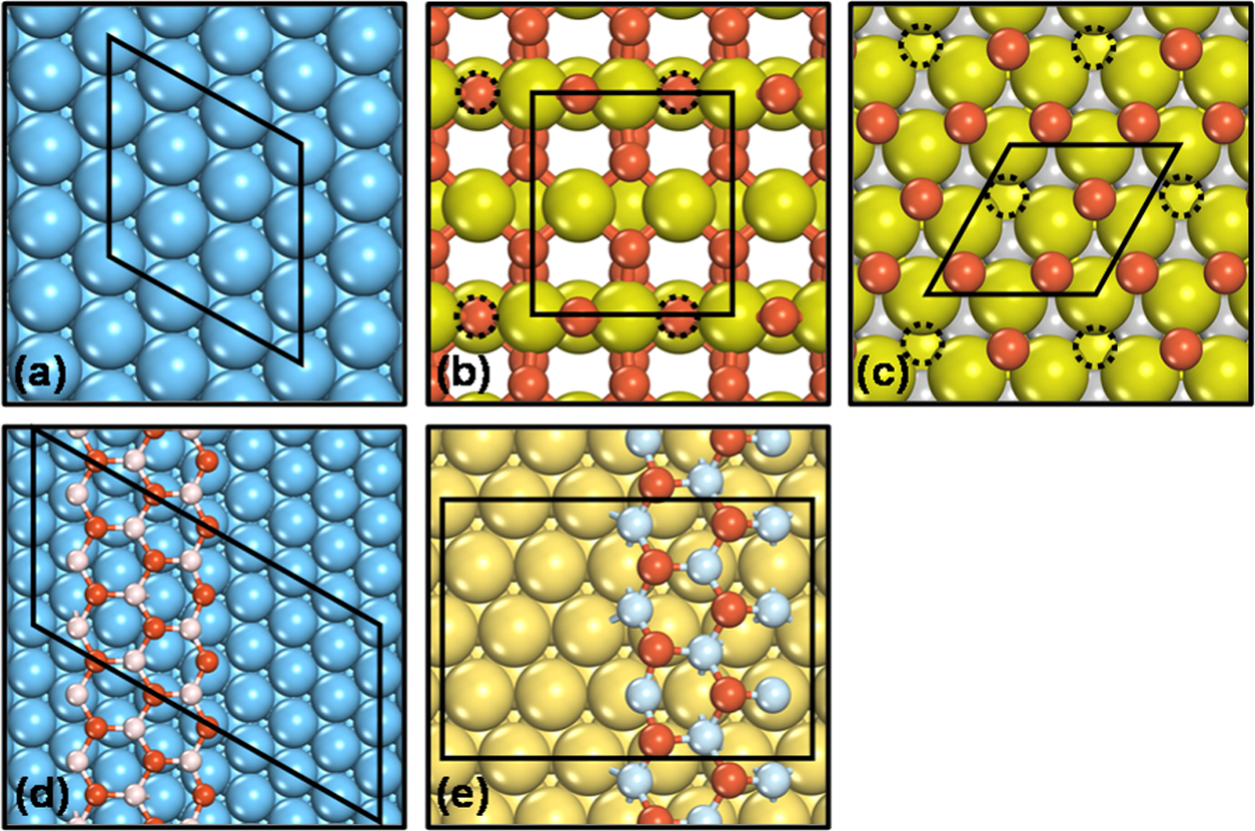
Top view of (a) Cu(111), (b) TiO_2_(110), (c) Ti_3_C_2_O_2_(0001), (d) Cu/ZnO, and (e) Pt/FeO. Blue, olive, pink, yellow, light blue, and red balls and black dashed circles represent Cu, Ti, Zn, Pt, Fe, and O atoms and O vacancies, respectively.

Cu(111), Co(0001), and Pt(111) were modeled using four-layer slabs with (3 × 3) surface unit cells (Figure 1a). The surface Brillouin zones were sampled with (4 × 4 × 1) Monkhorst–Pack *k*-point grid meshes.^44^ The two topmost layers and the adsorbates were fully relaxed, and the remaining layers were fixed at the converged bulk positions. (2 × 1) Four-layer and five-layer slabs were used to model TiO_2_(110) and Ti_3_C_2_O_2_(0001) surface; see Figure 1b,c. The two topmost layers of TiO_2_(110) and all of the layers of Ti_3_C_2_O_2_(0001) together with the adsorbates were relaxed. The Brillouin zones were sampled with (4 × 4 × 1) and (5 × 5 × 1) Monkhorst–Pack grids.^44^ A one-layer graphite-like (3 × 3) ZnO(0001) ribbon, with an in-plane lattice of 3.30 Å, on a three-layer (4 × 8) Cu(111) slab was adopted to simulate the Cu/ZnO interface (Figure 1d). The Brillouin zone was sampled with a (1 × 2 × 1) Monkhorst–Pack grid. The two bottommost Cu layers and the four leftmost ZnO columns were frozen, while the remaining atoms in the metal slab and the oxide together with the adsorbates were relaxed. The Pt/FeO interface was modeled by a (2√3 × 5) rectangular supercell, including a bilayer FeO ribbon with three columns of Fe atoms and two columns of O atoms on a three-layer Pt(111) slab, as shown in Figure 1e. A single *k*-point located at (0.25, −0.25, 0) was used to sample the surface Brillouin zone. The Pt layers and the three rightmost FeO columns were frozen, while the remaining atoms in the oxide were relaxed together with the adsorbates. The DFT + *U* approach was used to correct the on-site Coulomb repulsion of 3d electrons of Zn and Fe atoms in the Cu/ZnO and Pt/FeO interfaces, with *U*–*J* values of 4.7 and 3.0 eV, respectively.^45,46^ A vacuum region of at least 15 Å sufficed to avoid interactions between periodically repeated slabs along the *z*-direction for all of the systems studied. More details about the models can be found in previous works.^47−49^

The adsorption energy (Δ*E*_Ads_) was calculated using H_2_O, CH_3_OH, OH, and H_2_ in the gas phase as reference states since they are reasonably well described within DFT.^50,51^ A lower (more negative) Δ*E*_Ads_ implies stronger binding, while a higher (more positive) Δ*E*_Ads_ implies weaker binding. All transition states (TSs) were located by the CI-NEB method,^36^ and saddle points were confirmed by vibrational frequency analysis. The relaxations stopped when the residual forces on each atom were smaller than 0.05 eV/Å. The elementary activation barrier (Δ*E*_Act_ = *E*_TS_ – *E*_IS_, where TS and IS stand for transition and initial states, respectively) and reaction energy (Δ*H = E*_FS_ – *E*_IS_, where FS stands for final state) were calculated with respect to the co-adsorbed states of the species on the surfaces (for instance, Δ*H*_H_2_O_ =E_*H+*OH_ – E_*H_2_O_). We decompose the overall activation energy into two parts, namely, a preconditioning barrier and a dissociation barrier: Δ*E*_Act_ = Δ*E*_1_ + Δ*E*_2_. We note that to univocally define the preconditioning state, the rotation of O–H bonds and their stretching need to be successive events. However, our CI-NEBs have no specific constraints along the reaction coordinate such that the end of a rotation coincides with the stretching of O–H bonds by no more than 0.09 Å for all of the molecules studied; see Δ*d*_1_ in Table S1. We evaluated the effect of such overlap between rotation and stretching on the energy of the preconditioning states Δ*E*_1_ (see Tables S2 and S3 and more details in Section S1) and found that the small variation observed in O–H bond distances (<0.09 Å) from the initial states to the preconditioning states does not change the main conclusions of the present analysis (Figure S1). Compared to Δ*d*_1_, the variation in the dissociating O–H bond distance between transition states and preconditioning steps (Δ*d*_2_) is significantly larger, falling in the range of 0.04–0.71 Å (Table S1). We also define the rotation angle (∠ABC) of the dissociating O–H bond from the initial to the preconditioning step in Figure S2 and Table S4 to better describe the rotation of O–H bonds.

The Bader charge analysis was performed using a grid-based weight method^37^ in which the expression for the fraction of space neighboring each grid point that flows to its neighbors is used as a weight for the discrete integration of functions over the Bader volume. In this context, a positive or negative charge means charge depletion or charge accumulation, respectively.

## 3 Results and Discussion

### 3.1 H_2_O, CH_3_OH, and OH Activation on Metal Surfaces

**Figure 2:**
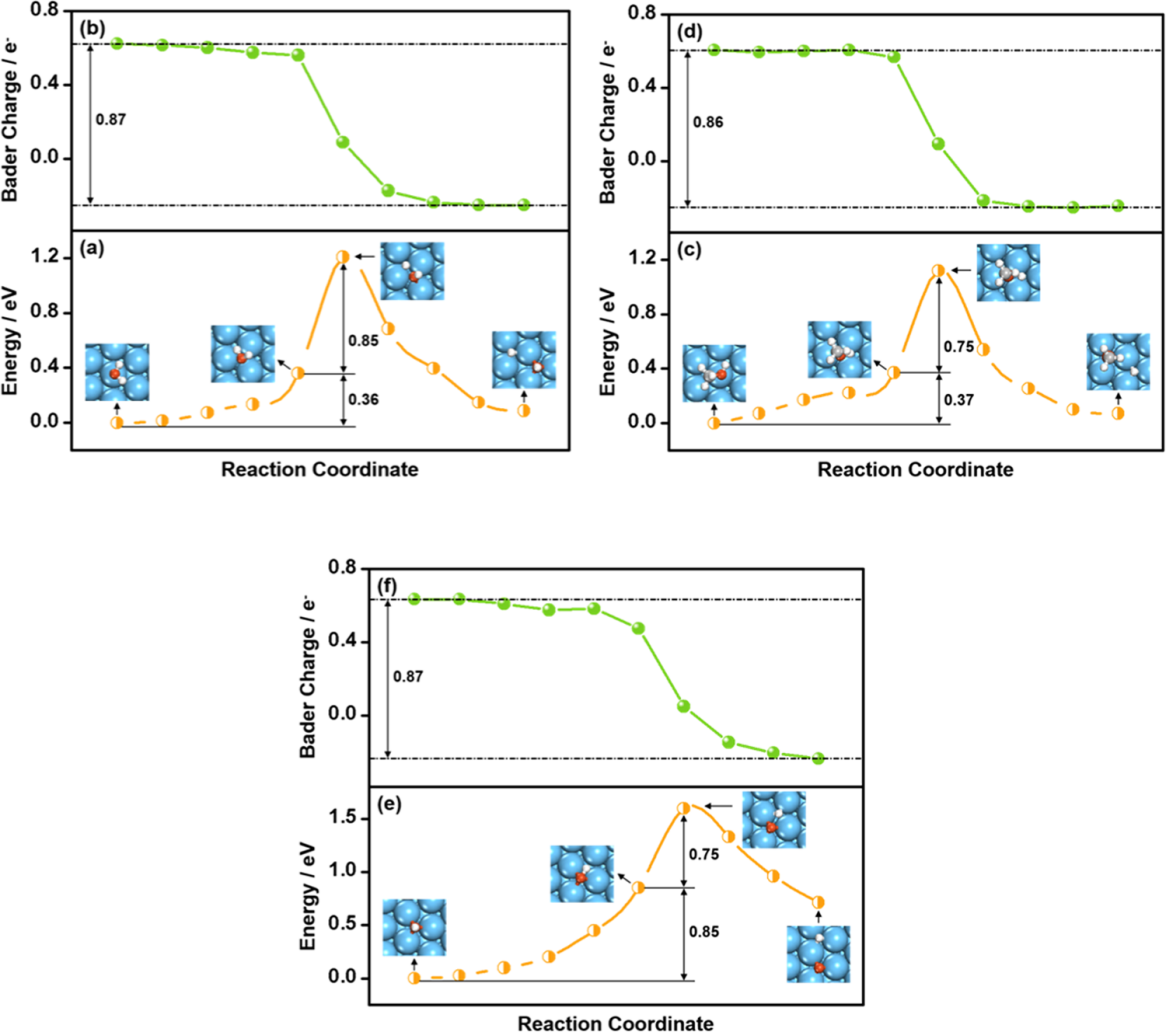
Energies and Bader charges on Cu(111) of a dissociating H moiety along the reaction coordinate for *H_2_O → *OH + *H (a, b); *CH_3_OH → *CH_3_O + *H (c, d); and *OH → *O + *H (e, f). Insets: snapshots of the initial, preconditioning, transition, and final states.

H_2_O binds weakly through O (Figures 2a, S3a, and S4a) at atop sites, with a flat-lying geometry (O and H at nearly identical heights above the surface), on metal surfaces. The calculated Δ*E*_Ads_ are −0.17, −0.28, and −0.24 eV on Cu(111), Co(0001), and Pt(111), respectively, as listed in Table S5. Such atop sites have been experimentally identified at low coverage and low temperature (<20 K) by scanning tunneling microscopy on Pt(111)^52^ and Cu(110),^53^ and predicted with DFT calculations to be the most stable adsorption sites on a number of close-packed and open metal surfaces.^54,55^ This is because the dipole moment of H_2_O molecules at these sites is aligned almost parallel to the surface plane, which favors the interaction of the 1b_1_ molecular orbital of H_2_O with the surface bands.^56^

Taking adsorbed H_2_O as the initial state (IS), we studied its dissociation on Cu(111), Co(0001), and Pt(111); see Table S6 and Figures 2a, S3a, and S4a. According to Table S6, the activation energies (Δ*E*_Act_) fall in the range of 0.88–1.21 eV, in agreement with previous DFT studies.^19,57−59^ These results indicate that H_2_O dissociation is difficult on metal surfaces at low temperatures. To rationalize these results, we analyzed how the energies and Bader charges of the dissociating H moiety change along the reaction coordinate on Cu(111).

The process begins with O moving from the top site to the hcp site and a rotation of the O–H bond. The rotation angles (∠ABC) of the dissociating O–H bond from the initial to the preconditioning state can be found in Figure S2 and Table S4. As a result, the water molecule is closer to the surface (generally by about 0.45 Å) with respect to the IS, and the O and H atoms differ in height above the surface. As shown in Table S1 and Figure 2a,b, the movement and rotation do not cause large variations of the O–H bond lengths (0.03 Å), energies (0.36 eV), and Bader charges of the dissociating H moiety (0.06 *e*^–^). However, as the H–OH distance increases to 1.44 Å at the TS, the H moiety breaks its bond with O in H_2_O and moves to an adjacent fcc site on Cu(111), with a net energy increase of 0.85 eV. Figure 2b shows that H exists in the form of a proton in adsorbed H_2_O, with a Bader charge of 0.62–0.56 *e*^–^. It becomes atomic H (0.09 to −0.25 *e*^–^) on Cu(111) after the O–H bond is broken. This suggests that H_2_O dissociation on Cu(111) is a H-like transfer process, where the H moiety in H_2_O needs 0.53 *e*^–^ (from 0.62 to 0.09 *e*^–^) to yield a H atom at the TS during dissociation, which entails a significant energy cost. This analysis also holds for Co(0001) and Pt(111), and explains the high activation energies (Δ*E*_Act_) for H_2_O dissociation on metals; see Figures S3 and S4.

CH_3_OH adsorption and activation are similar to those of H_2_O on metals. Briefly, CH_3_OH also binds through O in an atop configuration (Figures 2c, S3c, and S4c), with Δ*E*_Ads_ comparable to H_2_O on Cu(111), Co(0001), and Pt(111), as listed in Table S5. O–H bond scission in CH_3_OH has a high Δ*E*_Act_ of 0.75–1.12 eV on metals (Table S6), implying that CH_3_OH dissociation is as difficult as H_2_O dissociation. As shown in Figure 2c, the reaction coordinate on Cu(111) proceeds through an initial O diffusion from the top site to the hcp site, accompanied by a rotation of the O–H and C–O bonds (Table S4). CH_3_OH moves closer to the surface (generally by about 0.61 Å) during the surface diffusion and rotation, with a small change in the O–H bond lengths (0.02 Å), energies (0.37 eV), and Bader charges of the dissociating H (0.04 *e*^–^); see Table S1 and Figure 2c,d. After that, the O–H distance increases appreciably (by 0.43 Å), and so does the energy (by 0.75 eV) until the TS is reached. At the TS, the O–H bond is broken with H now bound to Cu, and the Bader charge of the dissociating H is lowered by as much as 0.52 *e*^–^ relative to the IS. Therefore, similar to H_2_O dissociation, CH_3_OH dissociation on metal surfaces is a H-like transfer process requiring significant charge transfer and energy expenses.

Moreover, the reaction coordinate of OH dissociation on Cu(111) is composed of a rotation of the O–H bond from an almost perpendicular configuration to one that is parallel to the surface plane (Figure S2 and Table S4), followed by its elongation. As shown in Figure 2e, rotating the O–H bond costs 0.85 eV on Cu(111), and its elongation to reach the TS costs 0.75 eV. This is clearly distinct from H_2_O and CH_3_OH dissociation, where the energy cost of the O–H bond stretching dominates Δ*E*_Act_. Compared with H_2_O and CH_3_OH, both having Δ*E*_Ads_ = −0.17 eV on Cu(111), *OH binds to the surface considerably stronger, with Δ*E*_Ads_ = −3.22 eV. According to valence shell electron pair repulsion theory,^60^ the stronger *OH binding should generally lead to O–H bond rotations with larger repulsion on the O–Cu bonds than in H_2_O and CH_3_OH. During O–H bond rotation, the Bader charge of the H moiety is only reduced by 0.16 *e*^–^ (Figure 2f). However, when the O–H bond is elongated and cleaved, the Bader charge of H is lowered by 0.43 *e*^–^. H-like transfers are also observed on Co(0001) and Pt(111), whereas the O–H bond rotation on Pt(111) is considerably easier than on Cu(111) and Co(0001) by 0.65 and 0.38 eV (Figures S3 and S4). This is because *OH binds atop on Pt(111) with the O–H bond tilted toward the surface plane (Figure S4e), in contrast with the adsorption at hollow sites observed on Cu(111) and Co(0001), where the O–H bond is perpendicular to the surface plane. We note in passing that the Δ*E*_Act_ and TS structures calculated here (Table S6 and Figures 2, S3, and S4) agree well with previous DFT studies.^13,19,57−59^

### 3.2 H_2_O, CH_3_OH, and OH Activation on TiO_2_(110) and Ti_3_C_2_O_2_(0001)

Compared to metal surfaces, the bridge O vacancy of TiO_2_(110) (Figure 1b) binds H_2_O and CH_3_OH more strongly, with Δ*E*_Ads_ of −0.90 and −0.99 eV, respectively (Table S5). These results can be rationalized considering the more significant electron donation from the lone-pair electrons of O (2p_*z*_) in H_2_O and CH_3_OH to the empty 3d states on TiO_2_(110) vs 3d and 4s states on Cu(111). This is seen from the deep-lying orbital hybridization in the energy window between −9 and −3 eV in Figure S5a,b. We attribute this to Ti atoms at the bridge O vacancy of TiO_2_(110) having more empty states than metallic Cu to accept lone-pair electrons.

**Figure 3:**
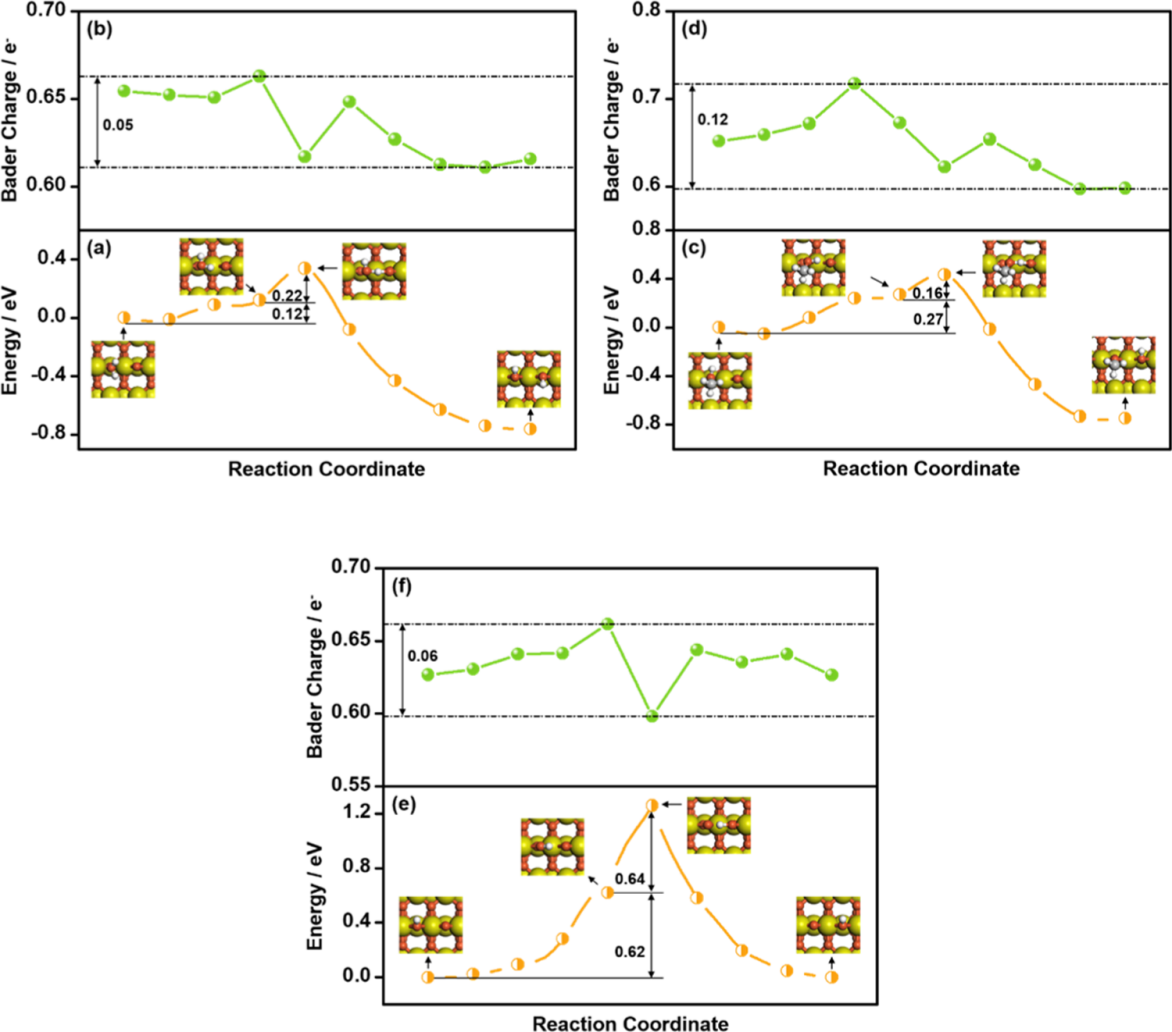
Energies and Bader charges on TiO_2_(110) of a dissociating H moiety along the reaction coordinate for *H_2_O → *OH + *H (a, b); *CH_3_OH → *CH_3_O + *H (c, d); and *OH + *O → *O + *OH (e, f). Insets: snapshots of the initial, preconditioning, transition, and final states.

Extracting H from the most stable states of adsorbed H_2_O at the bridge O vacancy to yield two adjacent bridge OH moieties on TiO_2_(110), has a reaction barrier of 0.34 eV (Table S6), in agreement with previous DFT studies.^61^ As shown in Figures 3a and S2 and Table S4, the reaction coordinate begins with water rotating to form a hydrogen bond with a neighboring bridge O site. Similar to the case of metals, the O–H bond rotation of adsorbed H_2_O only gives rise to a slight increase in energy (0.12 eV) and O–H bond length (0.02 Å); see Figure 3a and Table S1. After that, the O–H bond is elongated to 1.21 Å, and the TS is subsequently reached upon a small energy cost of 0.22 eV. This is noticeably different compared to metals, which have a substantial energy increase (0.85 eV on Cu(111) in Figure 2a) for the cleavage of the O–H bond in H_2_O.

Figure 3b shows that the Bader charge of H varies by no more than 0.05 *e*^–^ in the range of 0.61–0.66 *e*^–^ when it moves from H_2_O to O at the bridge site of TiO_2_(110). In view of this, H_2_O decomposition on TiO_2_(110) is a proton-like transfer process, does not involve a significant variation of electron charges and has low dissociation barriers, unlike the H-like transfer processes on metals. Similar to H_2_O dissociation, CH_3_OH dissociation on TiO_2_(110) is facile, with Δ*E*_Act_ = 0.44 eV (Table S6 and Figure 3c). Again, the low barrier is linked to a proton-like transfer during the dissociation process, wherein no significant change in the charge of the H moiety is noticed (Figure 3d).

*OH dissociation at the bridge O vacancy on TiO_2_(110) has a considerably higher Δ*E*_Act_ (1.26 eV) than H_2_O and CH_3_OH dissociation; see Table S6 and Figure 3e. Since the charge of H only varies by 0.06 *e*^–^ along the reaction coordinate (Figure 3f), the high Δ*E*_Act_ must stem from other reasons. The reaction coordinate proceeds through an initial O–H bond rotation to form a hydrogen bond with an adjacent bridge O site (Table S4), with a large associated energy cost of 0.62 eV (Figure 3e). This is in contrast with H_2_O and CH_3_OH dissociation, where the rotation only increases the energy by 0.12–0.27 eV. We also observed a more difficult rotation of *OH compared to H_2_O and CH_3_OH on metals, which we attribute to the stronger OH adsorption compared with H_2_O and CH_3_OH adsorption (−4.86 vs −0.90 and −0.99 eV), resulting in O–H bond rotations with larger repulsion. According to Table S6 and Figures S3e and S4e, OH dissociation on TiO_2_(110) has a higher barrier than on Co(0001) and Pt(111) by 0.27–0.31 eV, indicating that TiO_2_(110) is not efficient for OH activation despite its enhancement of H_2_O and CH_3_OH activation.

With respect to TiO_2_(110), the binding energies of H_2_O and CH_3_OH on Ti_3_C_2_O_2_(0001) are slightly weaker by no more than 0.10 eV, whereas the binding energies of dissociated species such as *OH and *CH_3_O are considerably stronger by 0.34–0.46 eV; see Table S5. Accordingly, not only are H_2_O and CH_3_OH dissociations more exothermic on Ti_3_C_2_O_2_(0001) than on TiO_2_(110), but Δ*E*_Act_ also decreases by 0.21–0.30 eV on this MXene (Table S6 and Figure S6). However, the case is different for OH dissociation, which has Δ*E*_Act_ = 1.40 eV on Ti_3_C_2_O_2_(0001), slightly higher than on TiO_2_(110) by 0.14 eV. Again, the facile dissociation of H_2_O and CH_3_OH is linked to a proton-like transfer process, whereas strong OH adsorption (−5.20 eV) is responsible for the unfavorable dissociation activity on Ti_3_C_2_O_2_(0001), as discussed above for TiO_2_(110). Finally, we note that both TiO_2_(110) and Ti_3_C_2_O_2_(0001) bind OH at the bridge or hollow sites with rather negative adsorption energies, which leads to high dissociation barriers. However, oxides binding OH on top sites may display weaker adsorption energies and, thus, more facile O–H bond rotation and faster dissociation kinetics.

To close this section, we note that, in agreement with our observations, Chandler et al. found through a combination of kinetics experiments, infrared spectroscopy experiments, and DFT calculations that H-like and proton-like transfers lead to dissimilar activities for H_2_ dissociation on TiO_2_-supported Au catalysts. Specifically, the heterolytic H_2_ dissociation, resulting in a formal hydride adsorbed on Au sites and a proton bound to the support to produce a TiOH group (proton-like transfer), has a lower barrier than the homolytic H_2_ dissociation on Au sites (H-like transfer) by 0.46–0.57 eV.^62^

### 3.3 H_2_O, CH_3_OH, and OH Activation on Cu/ZnO and Pt/FeO Interfaces

**Figure 4:**
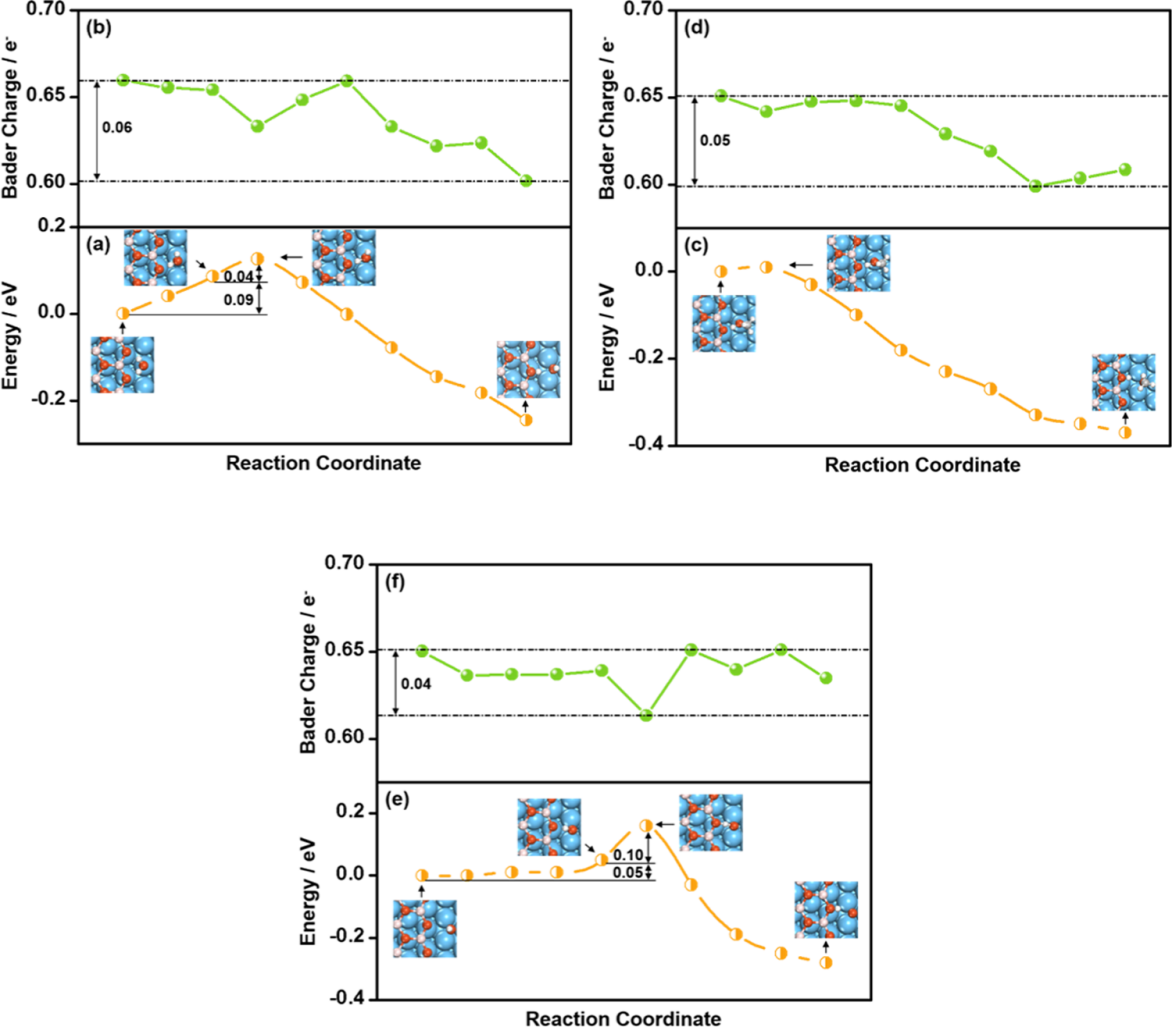
Energies and Bader charges on a Cu/ZnO interface of a dissociating H moiety along the reaction coordinate for *H_2_O → *OH + *H (a, b); *CH_3_OH → *CH_3_O + *H (c, d); and *OH + *O → *O + *OH (e, f). Insets: snapshots of the initial, preconditioning, transition, and final states.

We now turn our attention to metal/oxide interfaces, in particular Cu/ZnO and Pt/FeO. As shown in Figure 4, both H_2_O and CH_3_OH bind through O at the top sites of Cu atoms, with O–H bonds pointing to the O atom at the interface of Cu/ZnO (∠O–H–O = 154 and 160°; *d*_O–H_ = 1.76 and 1.41 Å), leading to the formation of strong hydrogen bonds. Such bonds stabilize H_2_O and CH_3_OH by 0.50 and 0.37 eV compared to Cu(111) (Table S5). *CH_3_O and *OH prefer to bind at the fcc site on the Cu terrace, with lower Δ*E*_Ads_ than on Cu(111) by ∼0.20 eV (Figures 4 and S7 and Table S5). OH is tilted toward the interfacial O atom on Cu/ZnO, in contrast to Cu(111), where the O–H bond is perpendicular to the surface plane. This implies that interfacial O atoms have an attractive interaction with H moieties in OH, which may facilitate the rotation of the O–H bond. H binds to the O atom at the interface between Cu(111) and ZnO (Figure S7), with a Δ*E*_Ads_ that is 0.63 eV more negative than on Cu(111). These adsorption properties result in more favorable thermochemistry for H_2_O, OH, and CH_3_OH dissociation on Cu/ZnO, with Δ*H* values from −0.24 to −0.37 eV (Table S6).

As shown in Figure 4, H_2_O, OH, and CH_3_OH dissociation on Cu/ZnO proceed through proton-like transfer processes, with a remarkably low Δ*E*_Act_ of 0.01–0.16 eV (Table S6). In particular, OH dissociation has Δ*E*_Act_ = 0.16 eV, which is substantially lower than those of metal and oxide surfaces (in the range of 0.95–1.60 eV, see Table S6). H_2_O and CH_3_OH form strong hydrogen bonds with interfacial O atoms upon adsorption at Cu/ZnO (Figure 4a,c), which avoid O–H bond rotation over wide angles (Figure S2 and Table S4) and their high associated energy costs. The rotation of the O–H bond in OH only costs 0.05 eV (Figure 4e), which is an order of magnitude lower than the corresponding values of 0.85, 0.62, and 0.68 eV on Cu(111), TiO_2_(110), and Ti_3_C_2_O_2_(0001). Compared to Cu(111), the inclination of OH toward an interfacial O atom at the IS and the formation of a hydrogen bond at the preconditioning state on the Cu/ZnO interface ease the rotation of O–H bonds. After the rotation, H–O–Cu bonds on the Cu/ZnO interface have an average angle of 104°, which is considerably larger than the 69° observed on Cu(111). Following valence shell electron pair repulsion theory,^60^ this results in lesser repulsion between O–H and O–Cu bonds and lower associated energy costs on the interface. In addition, the moderate binding of OH on the Cu/ZnO interface (Δ*E*_Ads_ = −3.41 eV) is considerably weaker than on TiO_2_(110) and Ti_3_C_2_O_2_(0001) by 1.45 and 1.79 eV (Table S5), which facilitates O–H bond rotation.

Besides O–H bond rotation, the proton-like transfer during H_2_O, OH, and CH_3_OH dissociation on Cu/ZnO is also facile, with energy costs of 0.10 eV or less (Figure 4). The O–H–O bond angles fall in the range of 160–168° after the O–H bond rotation during H_2_O, OH, and CH_3_OH dissociation, which are closer to the linear configuration than those on TiO_2_(110) and Ti_3_C_2_O_2_(0001) (130–141°). Furthermore, the O–H bond distances on Cu/ZnO are 1.29–1.59 Å after O–H bond rotation, which are shorter than the corresponding values on TiO_2_(110) and Ti_3_C_2_O_2_(0001) (1.68–1.89 Å). Such favorable configurations lead to stronger hydrogen bonds and proton-like transfer of H moieties with a concomitant low Δ*E*_Act_ (Figure 4).

The Fe-terminated Pt/FeO interface has a strong oxygen affinity, evinced by its OH and CH_3_O binding energies (Table S5). In fact, they are more negative than those of Pt(111) by 0.77 and 0.68 eV. However, atomic O binds at the Pt/FeO interface more weakly than on Pt(111) by 0.59 eV. This is because atomic O is only coordinated to an Fe atom at the interface, while three Pt atoms are available on Pt(111) (Figure S7Ca,Ga). In addition, H_2_O, CH_3_OH, and H at the interface have comparable Δ*E*_Ads_ to Pt(111). These results indicate that H_2_O and CH_3_OH dissociation are thermodynamically more favorable on Pt/FeO interfaces than on Pt(111) (Table S6), whereas the case is markedly different for OH dissociation.

As shown in Table S6 and Figure S8a–d, H_2_O and CH_3_OH dissociation proceed at the Pt/FeO interface through a proton-like transfer process with Δ*E*_Act_ values of 0.59 and 0.55 eV, which are lower than on Pt(111) (via a H-like transfer) by 0.33 and 0.32 eV, respectively. However, compared to other proton-like transfer processes on TiO_2_(110), Ti_3_C_2_O_2_(0001), and Cu/ZnO, the barriers are substantially higher. This may stem from the fact that after the O–H bond rotation, H_2_O and CH_3_OH binding at interfacial Fe sites cannot form hydrogen bonds as effectively as other oxides and metal/oxide interfaces, with O–H–O bond angles of 138 and 117° and O–H distances of 1.60 and 1.91 Å, respectively.

Unlike H_2_O and CH_3_OH dissociation, OH dissociation on Pt/FeO has a large barrier of 3.27 eV. Besides the unfavorable thermochemistry (Δ*H* = 1.29 eV), H-like transfers during OH dissociation at the Pt/FeO interface are observed from the IS to the TS and from there to the FS. According to Figure S8e,f, H binds to one Fe atom at the TS, with a variation in charge of 0.62 *e*^–^ with respect to the IS (H-like transfer). This differs from the proton-like transfer for H_2_O and CH_3_OH dissociation on Pt/FeO and can be understood from the adsorbate-interface structure. Each interfacial O binds to three Fe atoms and only one sp^3^ hybridization orbital perpendicular to the surface is available to bind the dissociated H moieties. Compared to interfacial O, the O atoms in adsorbed H_2_O and CH_3_OH are higher by 0.75 and 1.28 Å, whereas that in OH is lower by 0.31 Å. This leads to a less effective overlap between O (adsorbate)–O (interface) orbitals in OH compared to H_2_O and CH_3_OH and, hence, unfavorable transfer of H moieties.

In Sections 3.2 and 3.3, we showed that hydrogen bonding plays an important role in O–H scission on oxides, MXenes, and metal/oxide interfaces. Its effect is twofold: (1) H_2_O and CH_3_OH first undergo an O–H bond rotation to form a hydrogen bond with a neighboring O at the preconditioning states of oxidized surfaces and interfaces. This often leads to an energy stabilization of 0.20–0.30 eV^63,64^ and smaller energetic costs for O–H bond rotation. For instance, the preconditioning barriers Δ*E*_1_ for H_2_O/CH_3_OH rotation on the oxidized surfaces and interfaces fall in the range of 0.01–0.16/0.00–0.27 eV, which are lower than those of metals (0.20–0.46/0.30–0.37 eV; see Table S7). (2) Hydrogen bonds facilitate proton-like transfers, which are less energy-demanding than the H-like transfers observed on metals. For example, the dissociation barriers Δ*E*_2_ for H_2_O and CH_3_OH in Table S7 for oxidized surfaces and interfaces (0.01–0.43 eV) are appreciably lower than those of metals (0.42–0.85 eV).

Moreover, increasingly strong hydrogen bonding on oxidized surfaces and interfaces accelerates proton-like transfers. For instance, the Cu/ZnO interface with more linear O–H–O bond angles and shorter O–H distances (i.e., stronger hydrogen bonding) in the preconditioning steps for H_2_O, CH_3_OH, and OH scission has Δ*E*_2_ = 0.04, 0.01, and 0.10 eV, lower than the corresponding values of the other oxidized surfaces and interfaces (0.12–0.43, 0.13–0.43. and 0.64–0.71 eV, respectively; see Table S7).

### 3.4 Systematic Trends in the Scission of O–H Bonds

**Figure 5:**
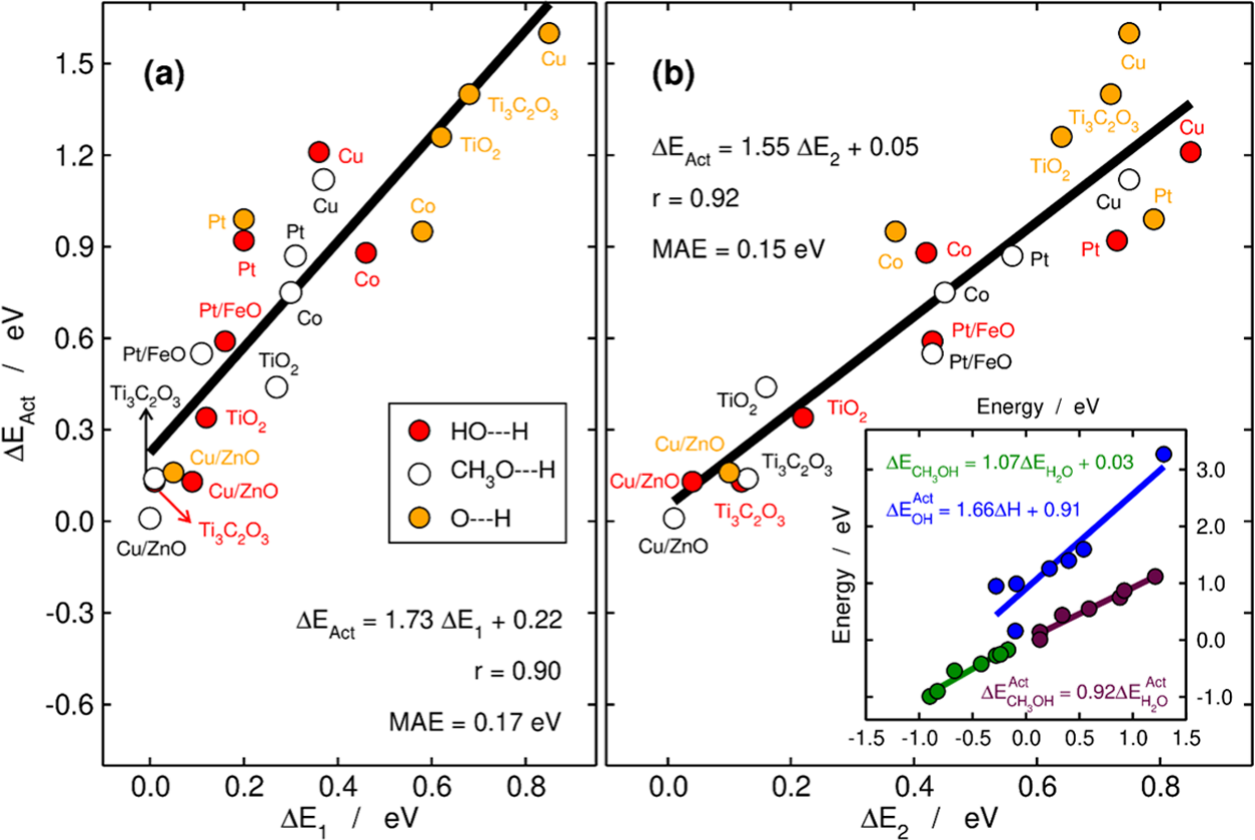
Trends in the kinetics and thermodynamics of H_2_O (red), CH_3_OH (white), and OH (orange) catalytic dissociation. (a) Overall activation energy (Δ*E*_Act_) as a function of the preconditioning barrier (Δ*E*_1_). (b) Overall activation energy as a function of the dissociation barrier (Δ*E*_2_). The equations of the linear fits are provided in each case together with the correlation coefficients (*r*) and the associated mean absolute errors (MAEs). Inset: Brønsted–Evans–Polanyi (BEP) relationship for *OH scission (blue), correlations between the adsorption energies of H_2_O and CH_3_OH (green), and the activation energies of their scissions (maroon). The *r* values are 0.93, 0.98, and 0.98, and the MAEs are 0.27, 0.04, and 0.05 eV, respectively.

Beyond the case-by-case analysis in Figures 2–4, S3, S4, S6, and S8, it is possible to extract overall trends from the data in this study. Δ*E*_Act_ can be split into two parts, namely, a preconditioning barrier and a dissociation barrier (hereon denoted as Δ*E*_1_ and Δ*E*_2_, respectively; see Table S7; see also Sections 2 and S1 for the determination of preconditioning states). Δ*E*_1_ is mostly related to the rotation of the O–H bonds, whereas Δ*E*_2_ corresponds to their actual cleavage. Figure 5 shows that Δ*E*_Act_, Δ*E*_1_, and Δ*E*_2_ are approximately correlated in a linear manner, implying that the costs of rotating and cleaving O–H bonds are adsorbate- and materials-specific yet proportional. All in all, lower Δ*E*_Act_ is consistently observed on oxidized materials and metal/oxide interfaces compared to metals. In addition, it is generally easier to cleave water and methanol than OH, except for Cu/ZnO interfaces, which cleave the three adsorbates with equally low barriers.

Furthermore, the inset in Figure 5b (blue line) shows that Brønsted–Evans–Polanyi (BEP) relations^65−67^ hold for OH dissociation on the materials under study. These relations connect a thermodynamic variable (Δ*H*) easy to calculate using DFT with a kinetic variable (Δ*E*_Act_) obtained through complicated transition-state searches. Although BEP relations are not observed for the activation of water and methanol, the inset in Figure 5 shows that their adsorption energies are proportional and so are the activation energies of their dissociation. Besides, Figures S9–S11 show that the adsorption energies of H_2_O and CH_3_OH, the total change in Bader charge (ΔBC) from the initial to the transition state, ΔBC^2^, and the geometric mean of the Bader charges between the initial and transition states (denoted as *G*(BC_IS_, BC_TS_)) are well correlated with Δ*E*_Act_. In addition, since the Bader charges at the transition states might be difficult to assess, we found a correlation between the mean Bader charges of initial and transition states and the Bader charges of the final states, as shown in Figure S12. In sum, Figures 5 and S9–S12 suggest that, in spite of the wide diversity of the materials under study, there are energetic and electronic descriptors that might be used to devise high-throughput routines to search for efficient catalysts to cleave methanol, water, and/or OH. Interestingly, a good catalyst for H_2_O activation is most certainly good for methanol activation, and vice versa. However, only in the presence of strong hydrogen bonding at interfaces might OH be inexpensively cleaved.

## 4 Conclusions

Knowledge of the underlying factors determining water and methanol activation is necessary for the design of enhanced catalysts for numerous reactions in catalysis. Finding such factors is usually complicated in view of the heterogeneity of the materials used to catalyze those processes and because of the great computational expenses associated with the assessment of kinetic barriers at surfaces and interfaces.

Here, through an interplay of CI-NEB transition-state searches and Bader charge analysis, we identified the key roles of proton-like transfer and O–H bond rotation in H_2_O, OH, and CH_3_OH activation on metals, oxides, MXenes, and metal/oxide interfaces. We provided a unifying framework for understanding the activation of O–H bonds, which allowed us to identify the active sites where it is more favorable, namely, at oxidized materials and metal/oxide interfaces, preferably offering strong hydrogen bonds. At those sites, O–H bond scission is accompanied by a proton-like transfer of H moieties and easy rotation of O–H bonds. Furthermore, we observed that an active material for cleaving water is likely suitable for methanol activation, but this need not be the case for OH dissociation.

The energetic proportionality between easy rotation and efficient O–H bond cleavage together with BEP and similar relations shown here might be used for the high-throughput in silico design of improved catalysts for reactions of industrial and technological interest in heterogeneous catalysis (e.g., methane/methanol steam reforming), electrochemistry (e.g., water splitting), and photocatalysis (e.g., methanol oxidation), where water, hydroxyl, and methanol are often present as reactants, intermediates, or products. In particular, the conspicuous differences between H_2_O/CH_3_OH and OH activation hint toward the use of carefully engineered multisite catalytic interfaces with controllable hydrogen bonding.

## Supporting Information Available

Determination of preconditioning states; energetic and geometric data for adsorbates and elementary reactions; energies and Bader charges of dissociating H moieties along the reaction coordinates; projected density of states; correlation between activation energies, adsorption energies and Bader charges for H_2_O and CH_3_OH cleavage; and converged Cartesian coordinates and electronic energies (PDF)
