# Supplementary material for: Finding Key Factors for Efficient Water and Methanol Activation at Metals, Oxides, MXenes, and Metal/Oxide Interfaces
Source: ACS Catal. 2022 Jan 5;12(2):1237–46. doi: 10.1021/acscatal.1c03405 (PMC8788388; doi:10.1021/acscatal.1c03405)
Supplement: Supplementary file 1 — cs1c03405_si_001.pdf [file cs1c03405_si_001.pdf]

Supporting Information

Finding Key Factors for Efficient Water and Methanol Activation at Metals, Oxides, MXenes and Metal/Oxide Interfaces

Hai-Yan Su,[a] Keju Sun,[b,] Xiang-Kui Gu,[c] Sha-Sha Wang,[c] Jing Zhu,[c] Wei-Xue Li,[c] Chenghua Sun,[d] and Federico Calle-Vallejo[e,*]*

[a] School of Chemical Engineering and Energy Technology, Dongguan University of Technology, Dongguan 523808, China.

[b] Key Laboratory of Applied Chemistry, College of Environmental and Chemical Engineering, Yanshan University, 438 Hebei Avenue, Qinhuangdao 066004, China.

[c] Department of Chemical Physics, College of Chemistry and Materials Science, Hefei National Laboratory for Physical Sciences at the Microscale, iChEM, CAS Center for Excellence in Nanoscience, University of Science and Technology of China, Hefei 230026, China.

[d] Centre for Translational Atomaterials, Swinburne University of Technology, Hawthorn, Victoria 3122, Australia.

[e] Department of Materials Science and Chemical Physics & Institute of Theoretical and Computational Chemistry (IQTCUB), University of Barcelona, Martí i Franquès 1, 08028 Barcelona, Spain.

**E-mail: kjsun@ysu.edu.cn (Keju Sun); f.calle.vallejo@ub.edu (Federico Calle-Vallejo)*

Table of contents

S1. Determination of preconditioning states 2

S2. Energetic and geometric data for adsorbates and elementary reactions 9

S3. Energies and Bader charges of dissociating H moieties along the reaction coordinate on Co(0001) and Pt(111) 11

S4. Projected density of states for H_2O and CH_3OH adsorption 13

S5. Energies and Bader charges of dissociating H moieties along the reaction coordinate on $Ti_3C_2O_2$(0001) 14

S6. Schematics of the converged geometries of the intermediates 15

S7. Energies and Bader charges of dissociating H moieties along the reaction coordinate on Pt/FeO 16

S8. Correlations between activation energies, adsorption energies, and Bader charges for H_2O and CH_3OH cleavage 17

S9. Dispersion effects on the adsorption energies of H_2O and CH_3OH 21

S10. Converged Cartesian coordinates and electronic energies 22

S11. References 28

S1. Determination of preconditioning states

To evaluate the effect of the overlap between O-H bond rotation and stretching on the energy of the preconditioning state, we calculated the total energies of gaseous H_2O, CH_3OH and OH with elongated O–H bonds (Δd_1 in Table S1) in the range of 0.01–0.09 Å, as in the corresponding preconditioning states. As listed in Table S2, the elongated O–H bonds only lead to small increases in the total energies ($\Delta E = E_{\text{elong}} - E$, where E_{elong} and E stand for the energy of molecules with elongated O–H bonds and at the DFT ground state) of gaseous H_2O, CH_3OH and OH by 0.01–0.08 eV, 0–0.14 eV, and 0.04–0.14 eV, respectively.

We then use the values of ΔE to correct the preconditioning barriers (ΔE_1 in Table S3), so that the energy variation among materials due to the different O–H bond stretching at the preconditioning states is considered. We define the stretch-corrected version of ΔE_1 (referred to as $\Delta E_{1,\text{C}}$ in Table S3) as:

$$\Delta E_{1,C} = \Delta E_1 - \Delta E \qquad \text{(S1)}$$

Taking H_2O on Cu(111) as an example, the O–H bond is stretched by 0.03 Å (Δd_1 in Table S3) at the preconditioning state with respect to the initial state. As listed in Table S2, the stretched O–H bond (0.03 Å) leads to an energy increase of gaseous H_2O by 0.02 eV. Hence, we have: $\Delta E_{1,\text{C}}$ = 0.36 – 0.02 = 0.34 eV (see Table S3).

As shown in Figure S1, upon the corrections the correlation between the overall ΔE_{Act} of O–H bond scissions and $\Delta E_{1,\text{C}}$ persists and the trends are similar to those in Figure 5a in the main text. In addition, the inset of Figure S1 shows that the energies of the preconditioning state with and without corrections are strongly correlated.

Table S1. Variations of the dissociating O-H bond distance between preconditioning and initial states (Δd_1), between transition and preconditioning states (Δd_2), and between transition and initial states ($\Delta d_{Tot} = \Delta d_1 + \Delta d_2$) for the cleavage of H_2O, CH_3OH, and OH. All bond distances are given in Å.

adsorbate	material	Δd_1	Δd_2	Δd_{Tot}
H_2O	Cu(111)	0.03	0.43	0.46
	Co(0001)	0.03	0.28	0.31
	TiO_2(110)	0.02	0.20	0.23
	Cu/ZnO	0.05	0.10	0.15
	$Ti_3C_2O_2$(0001)	0.03	0.17	0.20
	Pt/FeO	0.05	0.30	0.35
	Pt(111)	0.06	0.71	0.77
CH_3OH	Cu(111)	0.02	0.43	0.46
	Co(0001)	0.01	0.29	0.30
	TiO_2(110)	0.03	0.21	0.24
	Cu/ZnO	0.00	0.04	0.04
	$Ti_3C_2O_2$(0001)	0.02	0.19	0.21
	Pt/FeO	0.01	0.28	0.30
	Pt(111)	0.08	0.52	0.60
OH	Cu(111)	0.09	0.49	0.58
	Co(0001)	0.05	0.31	0.36
	TiO_2(110)	0.04	0.26	0.30
	Cu/ZnO	0.06	0.20	0.26
	$Ti_3C_2O_2$(0001)	0.04	0.28	0.32
	Pt(111)	0.06	0.53	0.59

Table S2. Variation of the total energies of gaseous H_2O, CH_3OH and OH ($\Delta E = E_{elong} - E$, where E_{elong} and E stand for the energy of molecules with elongated O–H bonds and at the DFT ground state) owing to the elongation of O–H bonds (Δd_1) in the corresponding preconditioning states. All energies and bond distances are given in eV and Å, respectively. Note that only certain elongations are observed for each species on the catalysts under study, such that there are missing values in the last three columns.

Δd_1	ΔE (H_2O)	ΔE (CH_3OH)	ΔE (OH)
0.01	–	0.00	–
0.02	0.01	0.01	–
0.03	0.02	0.02	–
0.04	–	–	0.04
0.05	0.06	–	0.05
0.06	0.08	–	0.07
0.08	–	0.14	–
0.09	–	–	0.14

Table S3. Variations of the dissociating O–H bond distance between preconditioning and initial states (Δd_1), uncorrected (ΔE_1) and corrected ($\Delta E_{1,C}$) preconditioning barriers, and overall activation energies (ΔE_{Act}) for cleaving H_2O, CH_3OH, and OH. The energies and distances are in eV and Å, respectively. For systems with very small ΔE_1, such as $H_2O/Ti_3C_2O_2$, $CH_3OH/Ti_3C_2O_2$ and OH/Cu/ZnO, we set the corrected preconditioning barriers ($\Delta E_{1,C}$) to zero to avoid the appearance of slightly negative values.

adsorbate	material	Δd_1	ΔE_1	$\Delta E_{1,C}$	ΔE_{Act}
H_2O	Cu(111)	0.03	0.36	0.34	1.21
	Co(0001)	0.03	0.46	0.44	0.88
	TiO_2(110)	0.02	0.12	0.11	0.34
	Cu/ZnO	0.05	0.09	0.03	0.13
	$Ti_3C_2O_2$(0001)	0.03	0.01	0.00	0.13
	Pt/FeO	0.05	0.16	0.10	0.59
	Pt(111)	0.06	0.20	0.12	0.92
CH_3OH	Cu(111)	0.02	0.37	0.36	1.12
	Co(0001)	0.01	0.30	0.30	0.75
	TiO_2(110)	0.03	0.27	0.25	0.44
	Cu/ZnO	0.00	0.00	0.00	0.01
	$Ti_3C_2O_2$(0001)	0.02	0.01	0.00	0.14
	Pt/FeO	0.01	0.11	0.11	0.55
	Pt(111)	0.08	0.31	0.17	0.87
OH	Cu(111)	0.09	0.85	0.71	1.60
	Co(0001)	0.05	0.58	0.53	0.95
	TiO_2(110)	0.04	0.62	0.58	1.26
	Cu/ZnO	0.06	0.05	0.00	0.16
	$Ti_3C_2O_2$(0001)	0.04	0.68	0.64	1.40
	Pt(111)	0.06	0.20	0.13	0.99

Table S4. Rotation angle (∠ABC, see Figure S2) of the dissociating O-H bond from the initial to the preconditioning state for the cleavage of H_2O, CH_3OH, and OH on various materials.

adsorbate	material	∠ABC
H_2O	Cu(111)	62°
	Co(0001)	63°
	TiO_2(110)	51°
	Cu/ZnO	22°
	$Ti_3C_2O_2$(0001)	50°
	Pt/FeO	27°
	Pt(111)	40°
CH_3OH	Cu(111)	39°
	Co(0001)	77°
	TiO_2(110)	72°
	Cu/ZnO	0°
	$Ti_3C_2O_2$(0001)	8°
	Pt/FeO	36°
	Pt(111)	30°
OH	Cu(111)	98°
	Co(0001)	84°
	TiO_2(110)	72°
	Cu/ZnO	47°
	$Ti_3C_2O_2$(0001)	62°
	Pt(111)	43°

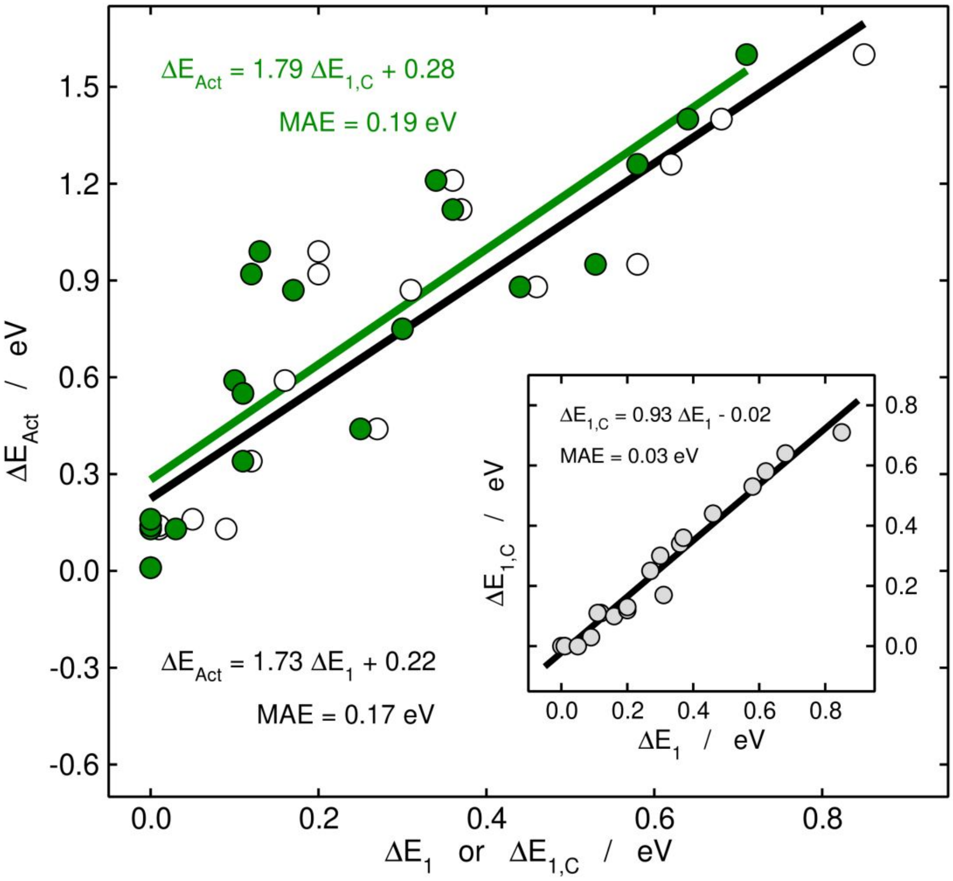

Figure S1. Overall activation energy (ΔE_{Act}) as a function of uncorrected (ΔE_1, black) and corrected preconditioning barriers ($\Delta E_{1,\mathrm{C}}$, green). Inset: corrected preconditioning barriers as a function of the uncorrected ones. In all cases, the equations of the linear fits and the mean absolute errors between the datapoints and the lines are provided.

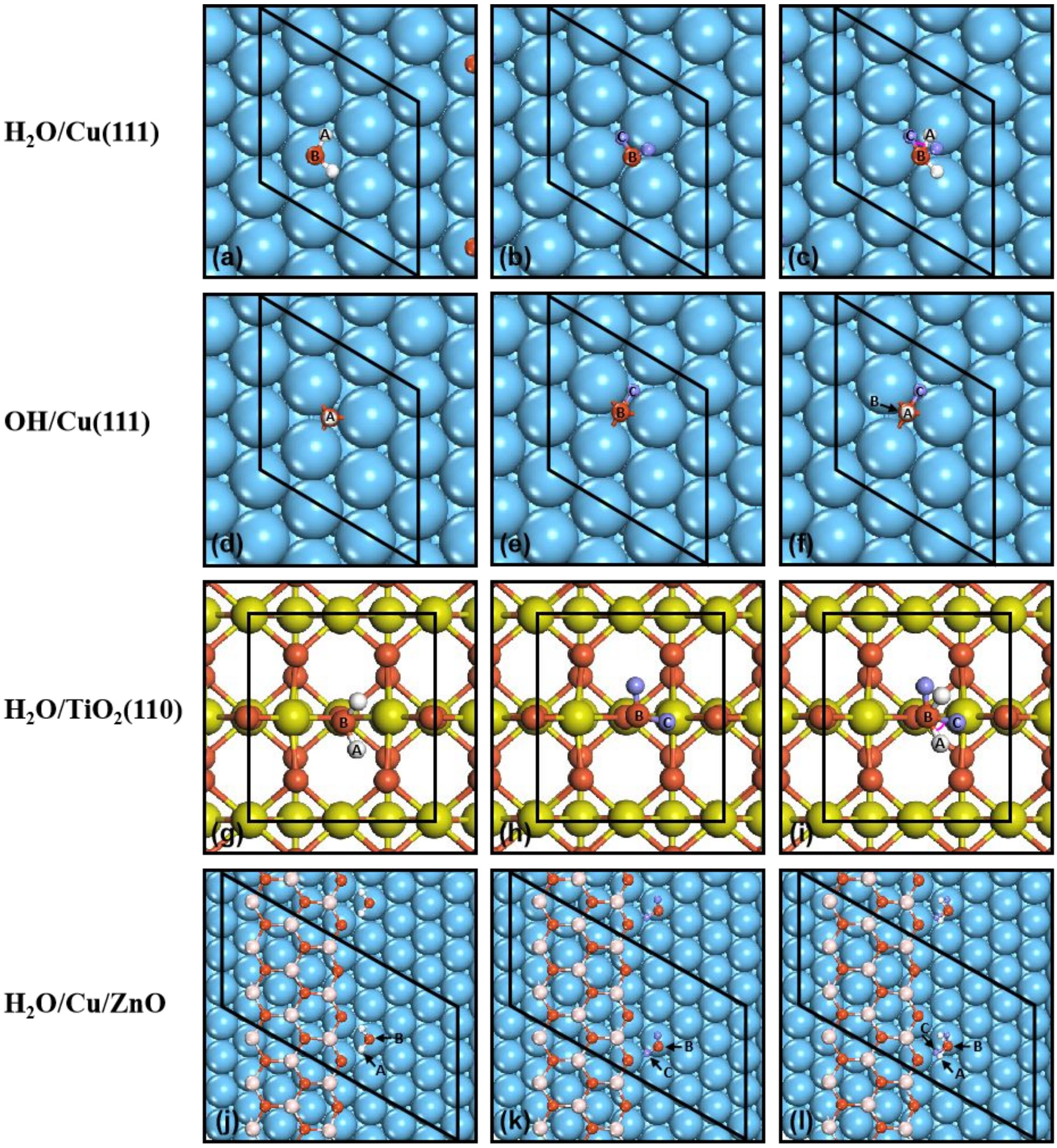

Figure S2. Structures of the initial state (a, d, g, j) and the preconditioning state (b, e, h, k) of H_2O and OH dissociation on Cu(111), TiO_2(110) and Cu/ZnO, and schematic diagram summarizing the O-H bond rotation from the initial to the preconditioning state during H_2O and OH dissociation (c, f, i, l). Note that H_2O and OH at the initial state are translated until their O atoms coincide with the O atoms at the preconditioning state. The blue, olive, pink, red, white, and purple balls represent Cu, Ti, Zn, O, H atoms at the initial and the preconditioning states, respectively. The ∠ABC is the rotation angle of the dissociating O-H bond and appears for each adsorbate and material in Table S4.

S2. Energetic and geometric data for adsorbates and elementary reactions

Table S5. Adsorption energies (ΔE_{Ads}, in eV) of various intermediates on Cu(111), Co(0001), Pt(111), TiO_2(110), $Ti_3C_2O_2$(0001), Cu/ZnO and Pt/FeO. The energies are calculated with respect to H_2, H_2O, OH and CH_3OH in the gas phase.

	Cu(111)	Co(0001)	Pt(111)	TiO_2(110)	$Ti_3C_2O_2$(0001)	Cu/ZnO	Pt/FeO
H_2O	-0.17	-0.28	-0.24	-0.90	-0.83	-0.67	-0.42
OH	-3.22	-3.65	-2.37	-4.86	-5.20	-3.41	-3.14
O	0.91	-0.08	1.34	-1.19	-1.49	0.70	1.93
H	-0.28	-0.53	-0.49	-0.13	0.00	-0.91	-0.51
CH_3OH	-0.17	-0.27	-0.25	-0.99	-0.90	-0.54	-0.42
CH_3O	0.08	-0.45	0.82	-1.67	-2.13	-0.13	0.14

Table S6. Activation energies (ΔE_{Act}, in eV), reaction energies (ΔH, in eV) and dissociated O-H bond lengths (d, in Å) at the TSs for various elementary reactions on Cu(111), Co(0001), Pt(111), TiO_2(110), $Ti_3C_2O_2$(0001), Cu/ZnO and Pt/FeO.

	$H_2O \rightarrow OH+H$			$CH_3OH \rightarrow CH_3O+H$			$OH \rightarrow O+H$		
	ΔE_{Act}	ΔH	d	ΔE_{Act}	ΔH	d	ΔE_{Act}	ΔH	d
Cu(111)	1.21	0.09	1.44	1.12	0.07	1.43	1.60	0.71	1.55
Co(0001)	0.88	-0.44	1.29	0.75	-0.52	1.29	0.95	-0.12	1.33
Pt(111)	0.92	0.71	1.75	0.87	0.66	1.58	0.99	0.01	1.57
TiO_2(110)	0.34	-0.76	1.21	0.44	-0.75	1.22	1.26	0.00	1.27
$Ti_3C_2O_2$(0001)	0.13	-1.19	1.18	0.14	-1.18	1.21	1.40	0.00	1.29
Cu/ZnO	0.13	-0.24	1.15	0.01	-0.37	1.11	0.16	-0.28	1.23
Pt/FeO	0.59	0.19	1.33	0.55	0.05	1.27	3.27	1.29	2.44

Table S7. Preconditioning barriers (ΔE_1), dissociation barriers (ΔE_2) and activation energies ($\Delta E_{Act} = \Delta E_1 + \Delta E_2$) for the cleavage of H_2O, CH_3OH, and OH. All energies are given in eV.

adsorbate	material	ΔE_1	ΔE_2	ΔE_{Act}
H_2O	Cu(111)	0.36	0.85	1.21
	Co(0001)	0.46	0.42	0.88
	TiO_2(110)	0.12	0.22	0.34
	Cu/ZnO	0.09	0.04	0.13
	$Ti_3C_2O_2$(0001)	0.01	0.12	0.13
	Pt/FeO	0.16	0.43	0.59
	Pt(111)	0.20	0.73	0.92
CH_3OH	Cu(111)	0.37	0.75	1.12
	Co(0001)	0.30	0.45	0.75
	TiO_2(110)	0.27	0.16	0.44
	Cu/ZnO	0.00	0.01	0.01
	$Ti_3C_2O_2$(0001)	0.01	0.13	0.14
	Pt/FeO	0.11	0.43	0.55
	Pt(111)	0.31	0.56	0.87
OH	Cu(111)	0.85	0.75	1.60
	Co(0001)	0.58	0.37	0.95
	TiO_2(110)	0.62	0.64	1.26
	Cu/ZnO	0.05	0.10	0.16
	$Ti_3C_2O_2$(0001)	0.68	0.71	1.40
	Pt(111)	0.20	0.79	0.99

S3. Energies and Bader charges of dissociating H moieties along the reaction coordinate on Co(0001) and Pt(111)

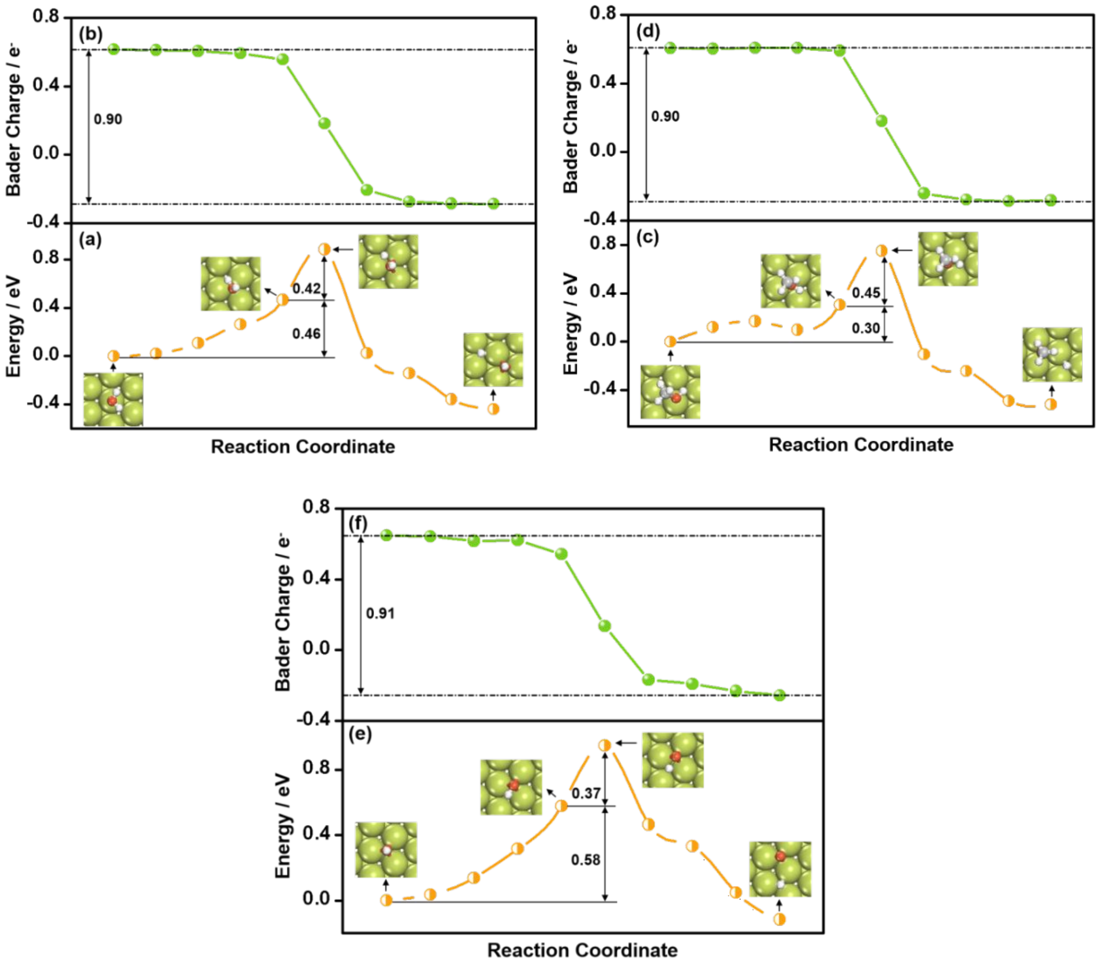

Figure S3. Energies and Bader charges of dissociating H moieties along the reaction coordinate on Co(0001) for $*H_2O \rightarrow *OH + *H$ (a, b); $*CH_3OH \rightarrow *CH_3O + *H$ (c, d); and $*OH \rightarrow *O + *H$ (e, f). Insets: snapshots of the initial, preconditioning, transition, and final states. The green, red, white, and grey balls represent Co, O, H and C atoms, respectively.

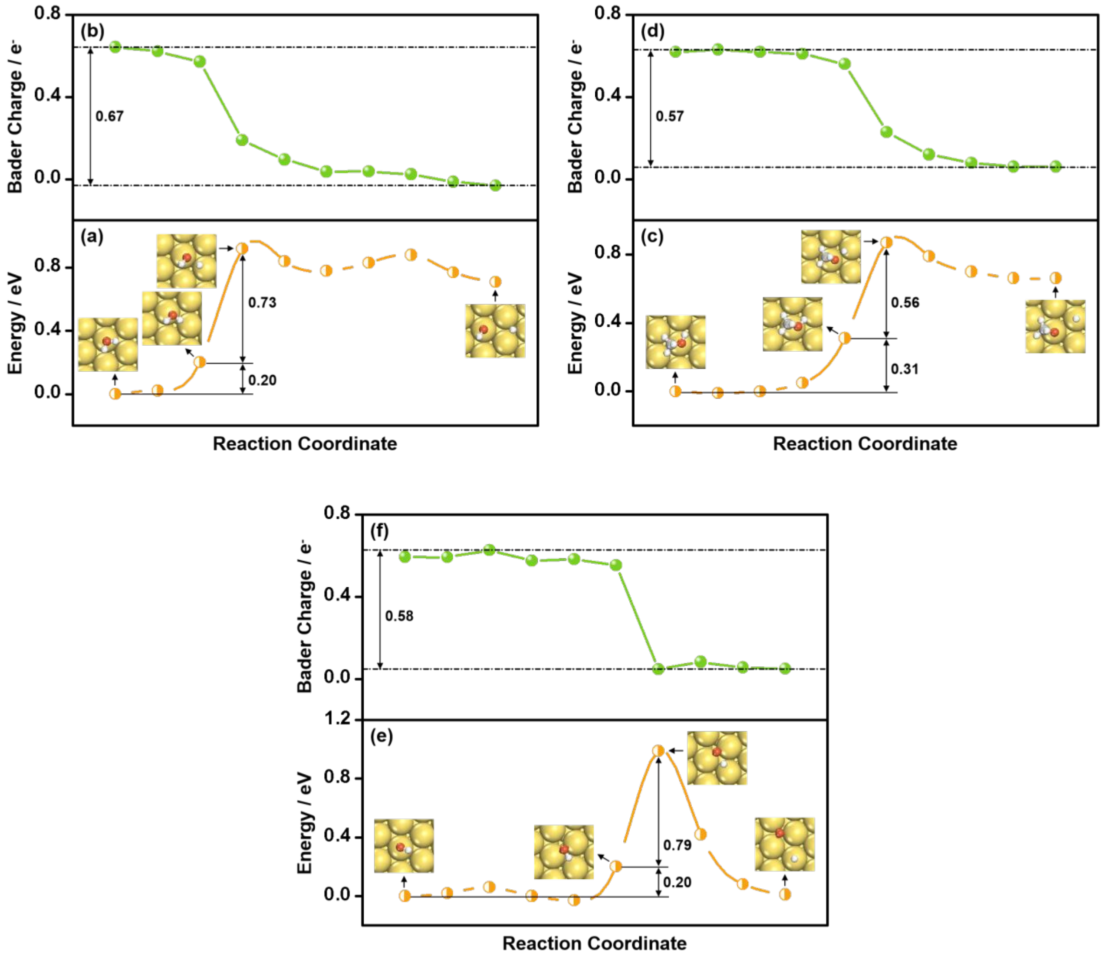

Figure S4. Energies and Bader charges of dissociating H moieties along the reaction coordinate on Pt(111) for $*H_2O \rightarrow *OH + *H$ (a, b); $*CH_3OH \rightarrow *CH_3O + *H$ (c, d); and $*OH \rightarrow *O + *H$ (e, f). Insets: snapshots of the initial, preconditioning, transition, and final states. The yellow, red, white, and grey balls represent Pt, O, H and C atoms, respectively.

S4. Projected density of states for H_2O and CH_3OH adsorption

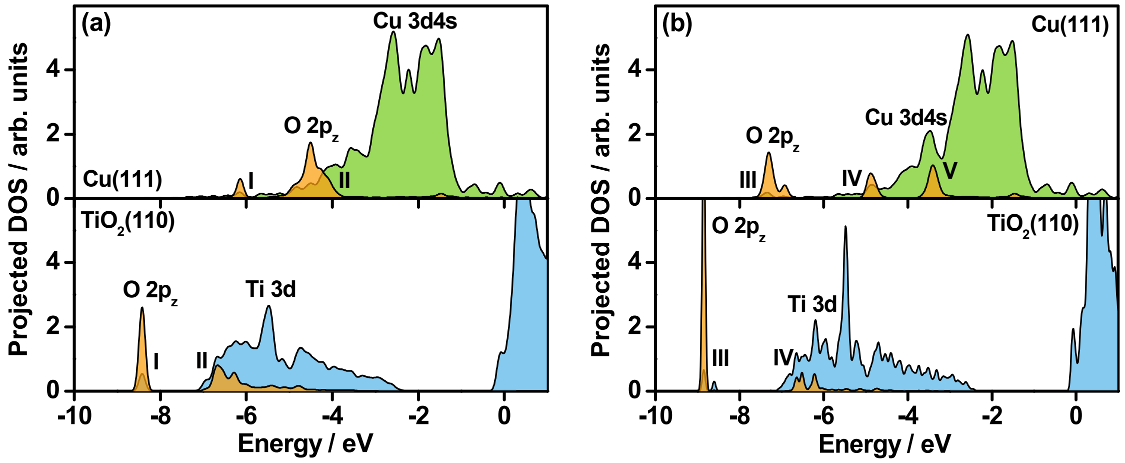

Figure S5. Projected density of states (PDOS) for (a) H_2O and (b) CH_3OH adsorption on Cu(111) and TiO_2(110). The 3*d* states of both Ti atoms at the bridge O vacancies and the 3*d* & 4*s* states of Cu atoms that bind to adsorbates are shown. PDOS of H_2O and CH_3OH adsorption on TiO_2(110) can be divided into two energy windows: [-9, -8] (I and III) and [-7, -6] (II and IV), which are lower at energies than those of [−6.5, -6] (I) and [−5, −4] (II) for H_2O/Cu(111) and those of [−7.5, −7] (III), [-5, -4.5] (IV) and [−4, −3] (V) for CH_3OH/Cu(111). Moreover, the deep-lying states on TiO_2(110) [-9, -8] have larger peak intensities than those on Cu(111), indicative of stronger hybridization between the two adsorbates and TiO_2(110).

S5. Energies and Bader charges of dissociating H moieties along the reaction coordinate on $Ti_3C_2O_2(0001)$

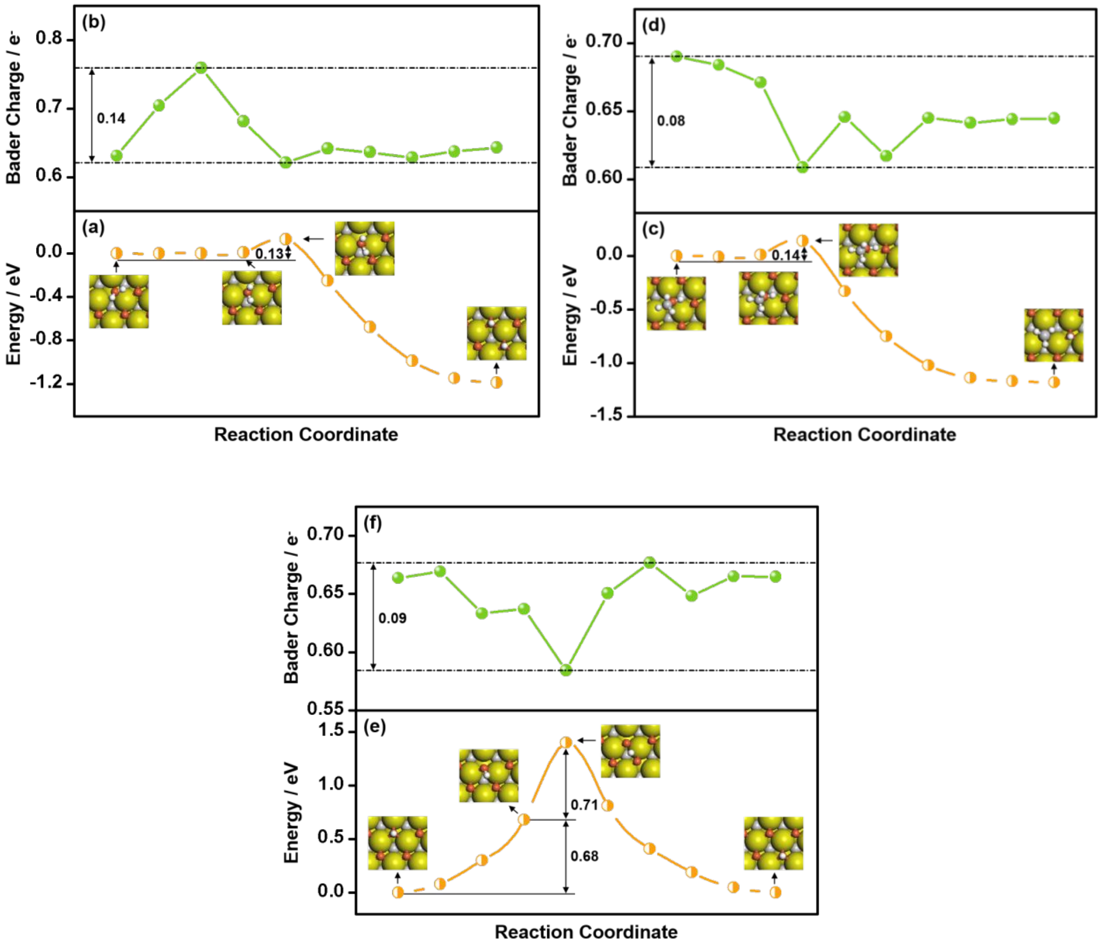

Figure S6. Energies and Bader charges on $Ti_3C_2O_2(0001)$ of dissociating H moieties along the reaction coordinate for $*H_2O \rightarrow *OH + *H$ (a, b); $*CH_3OH \rightarrow *CH_3O + *H$ (c, d); and $*OH + *O \rightarrow *O + *OH$ (e, f). Insets: snapshots of the initial, preconditioning, transition, and final states. The olive, red, white, and grey balls represent Ti, O, H and C atoms, respectively.

S6. Schematics of the converged geometries of the intermediates

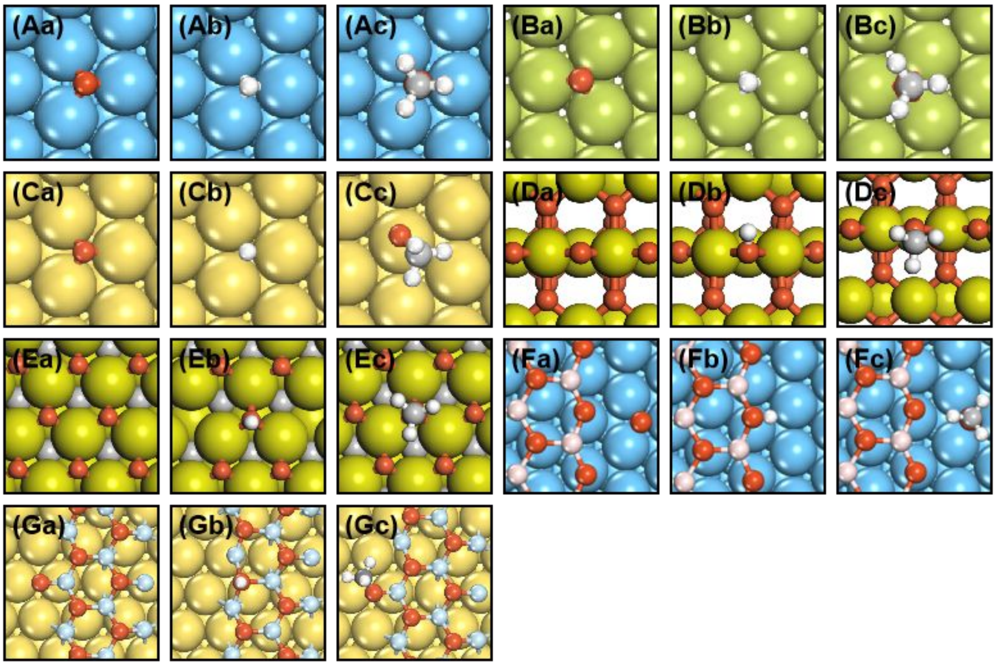

Figure S7. Optimized configurations for (a) O (b) H (c) CH_3O adsorption on Cu(111) (A), Co(0001) (B), Pt(111) (C), TiO_2(110) (D), $Ti_3C_2O_2$(0001) (E), Cu/ZnO (F) and Pt/FeO (G). The blue, green, yellow, olive, pink, light blue, red, white and grey balls represent Cu, Co, Pt, Ti, Zn, Fe, O, H and C atoms, respectively.

S7. Energies and Bader charges of dissociating H moieties along the reaction coordinate on Pt/FeO

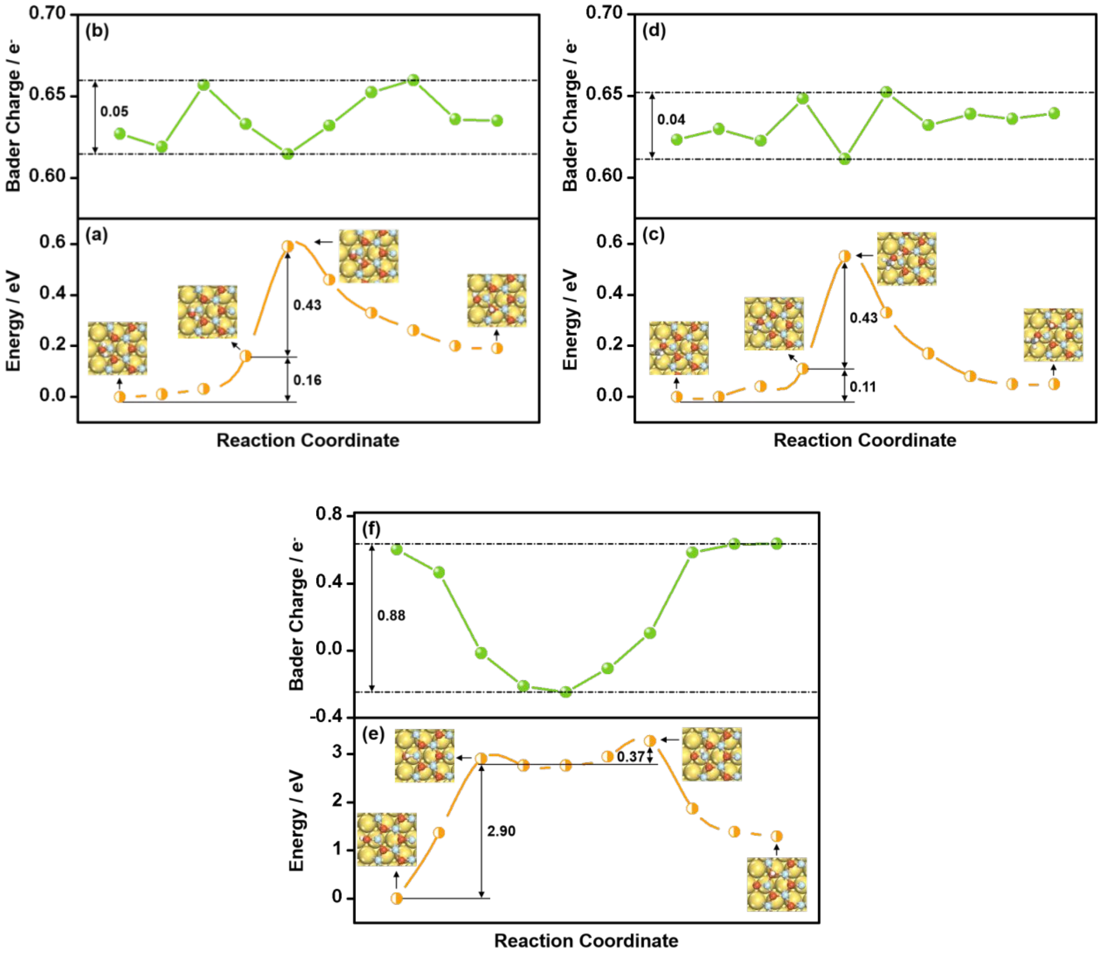

Figure S8. Energies and Bader charges on Pt/FeO of dissociating H moieties along the reaction coordinate for $*H_2O \rightarrow *OH + *H$ (a, b); $*CH_3OH \rightarrow *CH_3O + *H$ (c, d); and $*OH + *O \rightarrow *O + *OH$ (e, f). Insets: snapshots of the initial, preconditioning, transition, and final states. The yellow, light blue, red, white, and grey balls represent Pt, Fe, O, H and C atoms, respectively.

S8. Correlations between activation energies, adsorption energies, and Bader charges for H_2O and CH_3OH cleavage

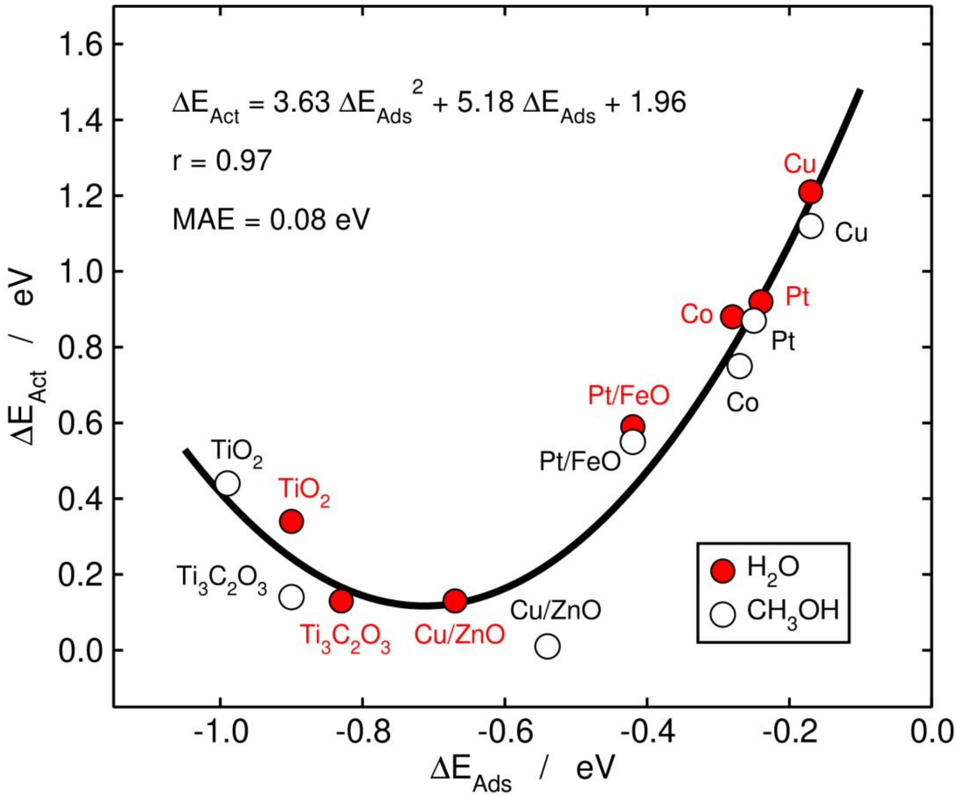

Figure S9. Trends in the activation energies (ΔE_{Act}) as a function of the adsorption energies of H_2O (red) and CH_3OH (white). The equation of the quadratic fit is provided in the figure together with the correlation coefficient (r) and the mean absolute error (MAE) between the fit and the calculated datapoints.

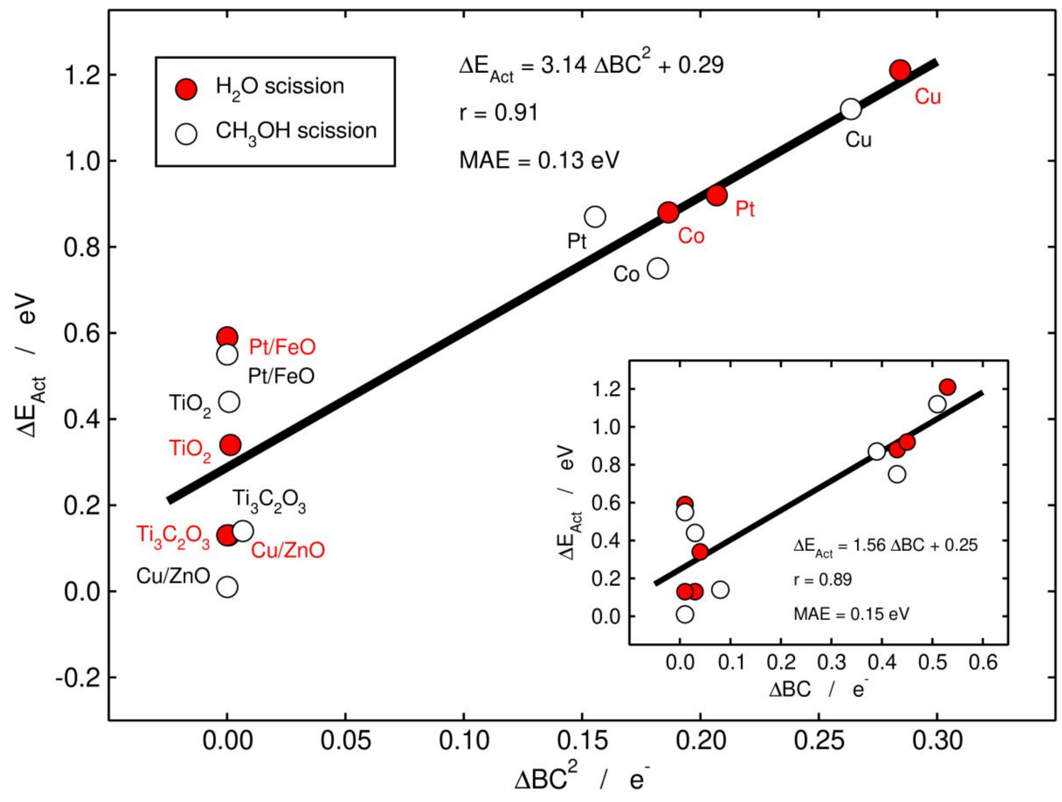

Figure S10. Correlation between the overall activation energies for H_2O (red) and CH_3OH (white) scissions and the squared difference between the Bader charges at the transition and initial states (ΔBC^2). Inset: Correlation between the overall activation energies and the difference between the Bader charges at the transition and initial states (ΔBC). The linear fits together with their correlation coefficient (r) and the mean absolute error (MAE) between the linear fit and the calculated datapoints are provided in each case.

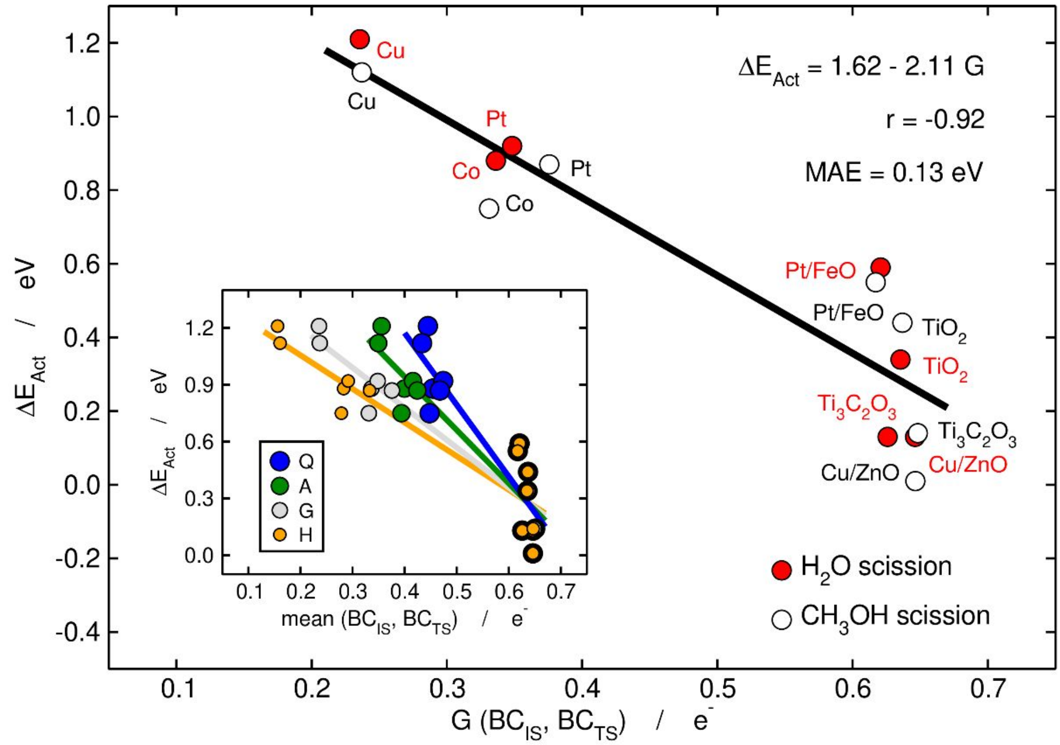

Figure S11. Correlation between the overall activation energies for H_2O (red) and CH_3OH (white) scissions and the geometric mean of the Bader charges at the transition and initial states (G (BC_{IS}, BC_{TS}). The linear fit together with its correlation coefficient (r) and the mean absolute error (MAE) between the linear fit and the calculated datapoints is provided. Inset: Correlation between the quadratic (Q), arithmetic (A), geometric (G), and harmonic (H) means of the Bader charges at the transition and initial states and the activation energies.

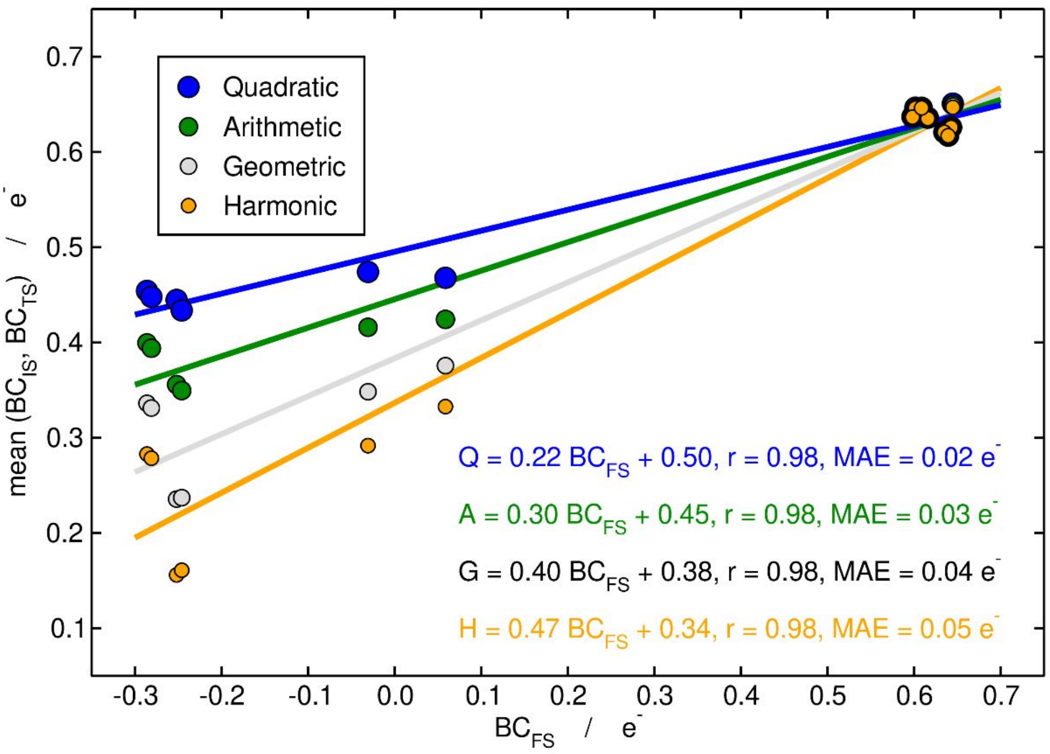

Figure S12. Correlation between the mean Bader charges at the transition and initial states (mean (BC_{IS}, BC_{TS}) and the Bader charges of the final states (BC_{FS}). The linear fits together with their correlation coefficients (r) and the mean absolute errors (MAE) between the linear fit and the calculated datapoints are provided in each case.

S9. Dispersion effects on the adsorption energies of H_2O and CH_3OH

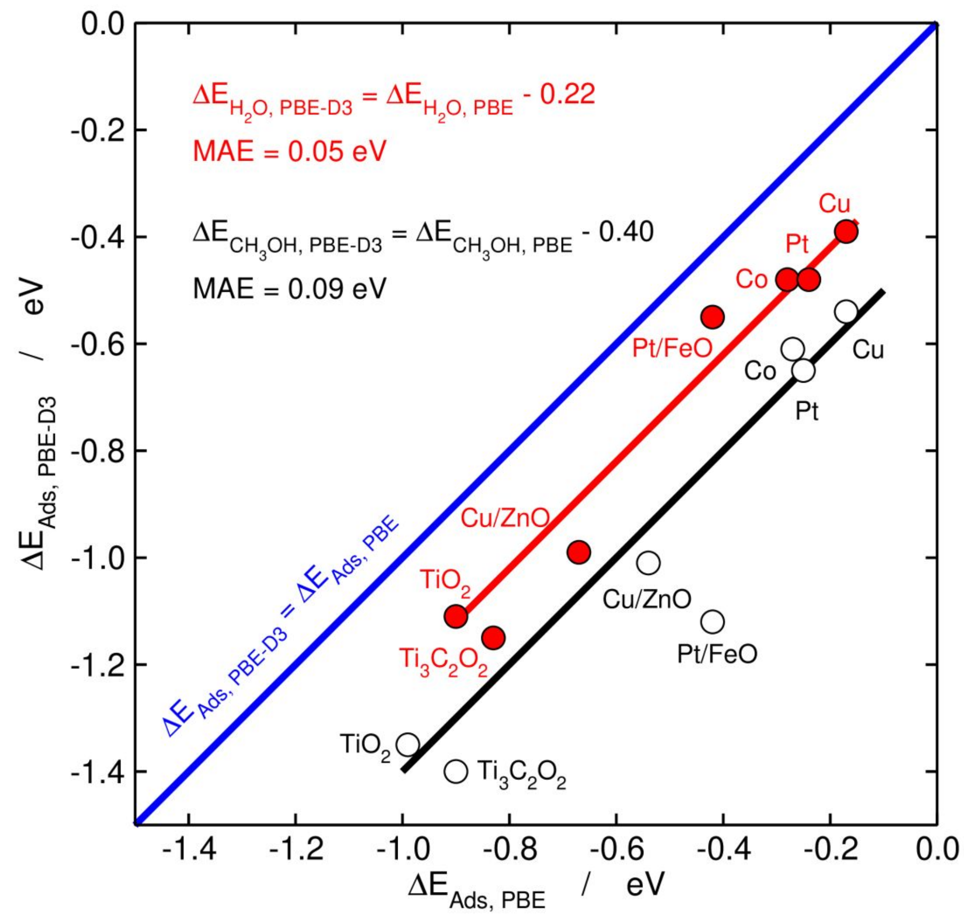

Figure S13. Parity plot showing the effect of D3 dispersion corrections with Becke-Johnson damping[1] on the adsorption energies of H_2O (red) and CH_3OH (white). The equations of the linear fits are provided in the figure together with the respective mean absolute errors (MAEs) between the fit and the calculated datapoints, assuming that the slopes are unity. D3 corrections shift downwards the PBE adsorption energies of water and methanol by 0.22 and 0.40 eV, respectively.

S10. Converged Cartesian coordinates and electronic energies

Initial state of *H_2O → *OH + *H on Cu(111) E=-139.87

Cu	0.000000	0.000000	0.000000
Cu	0.000000	2.571014	0.000000
Cu	0.000000	5.142106	0.000000
Cu	2.226564	-1.285507	0.000000
Cu	2.226564	1.285507	0.000000
Cu	2.226564	3.856599	0.000000
Cu	4.453196	-2.571053	0.000000
Cu	4.453196	-0.000039	0.000000
Cu	4.453196	2.571053	0.000000
Cu	0.742188	1.285507	2.099295
Cu	0.742188	3.856599	2.099295
Cu	0.742188	6.427613	2.099295
Cu	2.968753	0.000000	2.099295
Cu	2.968753	2.571091	2.099295
Cu	2.968753	5.142106	2.099295
Cu	5.195384	-1.285546	2.099295
Cu	5.195384	1.285546	2.099295
Cu	5.195384	3.856560	2.099295
Cu	1.483446	0.011407	4.199364
Cu	1.483734	2.562199	4.198461
Cu	1.474938	5.142137	4.166486
Cu	3.704058	-1.286256	4.173057
Cu	3.693553	1.287151	4.192328
Cu	3.702868	3.858313	4.171594
Cu	5.930396	-2.570055	4.178159
Cu	5.933941	0.003932	4.166027
Cu	5.934192	2.568510	4.167145
Cu	-0.018247	-0.003843	6.241448
Cu	-0.019659	2.578639	6.239685
Cu	-0.009342	5.143886	6.246356
Cu	2.220793	-1.297374	6.236871
Cu	2.213303	1.286148	6.336025
Cu	2.220677	3.872623	6.232775
Cu	4.442727	-2.570384	6.256450
Cu	4.455418	-0.007925	6.229294
Cu	4.456485	2.582623	6.230846
O	2.350149	1.341912	8.686036
H	2.781303	2.215372	8.782401
H	3.070814	0.693587	8.818397

Transition state of *H_2O → *OH + *H on Cu(111) E= -138.66

Cu	0.000000	0.000000	0.000000
Cu	0.000000	2.571014	0.000000
Cu	0.000000	5.142106	0.000000
Cu	2.226564	-1.285507	0.000000
Cu	2.226564	1.285507	0.000000
Cu	2.226564	3.856599	0.000000
Cu	4.453196	-2.571053	0.000000
Cu	4.453196	-0.000039	0.000000
Cu	4.453196	2.571053	0.000000
Cu	0.742188	1.285507	2.099295
Cu	0.742188	3.856599	2.099295
Cu	0.742188	6.427613	2.099295
Cu	2.968753	0.000000	2.099295
Cu	2.968753	2.571091	2.099295
Cu	2.968753	5.142106	2.099295
Cu	5.195384	-1.285546	2.099295
Cu	5.195384	1.285546	2.099295
Cu	5.195384	3.856560	2.099295
Cu	1.489200	-0.011935	4.163481
Cu	1.480132	2.574699	4.165072
Cu	1.500309	5.117767	4.177693
Cu	3.721293	-1.270059	4.208663
Cu	3.709160	1.275721	4.197624
Cu	3.715223	3.850379	4.185457
Cu	5.941755	-2.578078	4.176143
Cu	5.920285	0.000041	4.204833
Cu	5.934015	2.557923	4.173383
Cu	-0.004135	-0.016298	6.233308
Cu	-0.024618	2.575621	6.256032
Cu	0.045686	5.127475	6.229254
Cu	2.216149	-1.316813	6.232817
Cu	2.109666	1.276688	6.206926
Cu	2.251260	3.789871	6.330374
Cu	4.453808	-2.559080	6.253108
Cu	4.454706	-0.011943	6.365039
Cu	4.502103	2.619027	6.247681
O	3.854150	1.292085	7.945330
H	3.067885	2.358542	7.376805
H	4.252716	1.483983	8.821778

Final state of *H_2O → *OH + *H on Cu(111) E= -139.79

Cu	0.000000	0.000000	0.000000
Cu	0.000000	2.571014	0.000000
Cu	0.000000	5.142106	0.000000
Cu	2.226564	-1.285507	0.000000
Cu	2.226564	1.285507	0.000000
Cu	2.226564	3.856599	0.000000
Cu	4.453196	-2.571053	0.000000
Cu	4.453196	-0.000039	0.000000
Cu	4.453196	2.571053	0.000000
Cu	0.742188	1.285507	2.099295
Cu	0.742188	3.856599	2.099295
Cu	0.742188	6.427613	2.099295
Cu	2.968753	-0.000000	2.099295
Cu	2.968753	2.571091	2.099295
Cu	2.968753	5.142106	2.099295
Cu	5.195384	-1.285546	2.099295
Cu	5.195384	1.285546	2.099295
Cu	5.195384	3.856560	2.099295
Cu	1.493078	0.015564	4.179425
Cu	1.484957	2.579589	4.149322
Cu	1.475601	5.131027	4.201411
Cu	3.722411	-1.263109	4.188818
Cu	3.723374	1.288022	4.200126
Cu	3.723324	3.843163	4.182448
Cu	5.936335	-2.573244	4.164440
Cu	5.938819	0.016668	4.198123
Cu	5.914746	2.563303	4.211177
Cu	0.003117	0.022840	6.219354
Cu	-0.002397	2.541917	6.204499
Cu	0.108392	5.154445	6.303320
Cu	2.231006	-1.269903	6.225877
Cu	2.189597	1.291222	6.269721
Cu	2.224839	3.867969	6.267294
Cu	4.463630	-2.561996	6.227154
Cu	4.411660	-0.078463	6.309945
Cu	4.402837	2.647844	6.387916
O	5.274122	1.212479	7.610700
H	2.807969	2.606603	7.220160
H	5.282288	1.187702	8.583472

Initial state of *CH_3OH → *CH_3O + *H on Cu(111) E=-155.86

Cu	0.000000	0.000000	0.000000
Cu	0.000000	2.571014	0.000000
Cu	0.000000	5.142106	0.000000
Cu	2.226564	-1.285507	0.000000
Cu	2.226564	1.285507	0.000000
Cu	2.226564	3.856599	0.000000
Cu	4.453196	-2.571053	0.000000
Cu	4.453196	-0.000039	0.000000
Cu	4.453196	2.571053	0.000000
Cu	0.742188	1.285507	2.099295
Cu	0.742188	3.856599	2.099295
Cu	0.742188	6.427613	2.099295
Cu	2.968753	0.000000	2.099295
Cu	2.968753	2.571091	2.099295
Cu	2.968753	5.142106	2.099295
Cu	5.195384	-1.285546	2.099295
Cu	5.195384	1.285546	2.099295
Cu	5.195384	3.856560	2.099295
Cu	1.491572	0.002448	4.197545
Cu	1.491204	2.553493	4.196675
Cu	1.482857	5.132387	4.166249
Cu	3.713512	-1.291359	4.178232
Cu	3.700122	1.279591	4.198468
Cu	3.709245	3.852878	4.171325
Cu	5.937399	-2.577118	4.173168
Cu	5.940346	-0.002808	4.169693
Cu	5.939035	2.561371	4.164537
Cu	-0.008227	-0.014193	6.231330
Cu	-0.008807	2.567564	6.235388
Cu	0.002787	5.135092	6.247554
Cu	2.228114	-1.310292	6.235655
Cu	2.227242	1.270454	6.340807
Cu	2.231873	3.858647	6.239499
Cu	4.456325	-2.580957	6.254185
Cu	4.467313	-0.014318	6.240351
Cu	4.469373	2.576641	6.231424
C	1.257154	1.164151	9.494429
O	2.423399	1.310539	8.658693
H	0.820626	0.187651	9.245714
H	0.511503	1.947977	9.286811
H	1.542007	1.181867	10.557918
H	2.787273	2.212501	8.759763

Transition state of *CH_3OH → *CH_3O + *H on Cu(111) E= -154.74

Cu	0.000000	0.000000	0.000000
Cu	0.000000	2.571014	0.000000
Cu	0.000000	5.142106	0.000000
Cu	2.226564	-1.285507	0.000000
Cu	2.226564	1.285507	0.000000
Cu	2.226564	3.856599	0.000000
Cu	4.453196	-2.571053	0.000000
Cu	4.453196	-0.000039	0.000000
Cu	4.453196	2.571053	0.000000
Cu	0.742188	1.285507	2.099295
Cu	0.742188	3.856599	2.099295
Cu	0.742188	6.427613	2.099295
Cu	2.968753	-0.000000	2.099295
Cu	2.968753	2.571091	2.099295
Cu	2.968753	5.142106	2.099295
Cu	5.195384	-1.285546	2.099295
Cu	5.195384	1.285546	2.099295
Cu	5.195384	3.856560	2.099295
Cu	1.464958	0.005797	4.168212
Cu	1.472418	2.574552	4.199721
Cu	1.466301	5.142100	4.172759
Cu	3.694718	-1.281334	4.176101
Cu	3.702060	1.286404	4.172109
Cu	3.694035	3.861799	4.180402
Cu	5.934480	-2.552299	4.201968
Cu	5.936210	-0.008980	4.201323
Cu	5.897406	2.577552	4.179201
Cu	-0.039989	-0.018843	6.231431
Cu	-0.028506	2.571897	6.338303
Cu	-0.047637	5.183925	6.227632
Cu	2.200869	-1.278226	6.236231
Cu	2.253544	1.203054	6.227117
Cu	2.239327	3.946919	6.244755
Cu	4.449488	-2.553465	6.251348
Cu	4.453039	-0.005968	6.253692
Cu	4.362483	2.573998	6.338298
C	1.082687	2.850726	9.339237
O	1.326366	2.667656	7.935720
H	0.492959	2.003913	9.726850
H	0.522658	3.786141	9.504430
H	2.037382	2.903437	9.884702
H	2.647825	2.565940	7.390946

Final state of *CH_3OH → *CH_3O + *H on Cu(111) E= -155.79

Cu	0.000000	0.000000	0.000000
Cu	0.000000	2.571014	0.000000
Cu	0.000000	5.142106	0.000000
Cu	2.226564	-1.285507	0.000000
Cu	2.226564	1.285507	0.000000
Cu	2.226564	3.856599	0.000000
Cu	4.453196	-2.571053	0.000000
Cu	4.453196	-0.000039	0.000000
Cu	4.453196	2.571053	0.000000
Cu	0.742188	1.285507	2.099295
Cu	0.742188	3.856599	2.099295
Cu	0.742188	6.427613	2.099295
Cu	2.968753	0.000000	2.099295
Cu	2.968753	2.571091	2.099295
Cu	2.968753	5.142106	2.099295
Cu	5.195384	-1.285546	2.099295
Cu	5.195384	1.285546	2.099295
Cu	5.195384	3.856560	2.099295
Cu	1.499553	0.012752	4.178606
Cu	1.481985	2.577471	4.195469
Cu	1.487889	5.117668	4.213433
Cu	3.713220	-1.292946	4.164529
Cu	3.723362	1.283627	4.148644
Cu	3.690483	3.855653	4.177666
Cu	5.950732	-2.558299	4.189714
Cu	5.954703	0.005851	4.195196
Cu	5.937134	2.551654	4.200956
Cu	0.017718	0.006901	6.225265
Cu	-0.038608	2.497324	6.294414
Cu	-0.032774	5.230085	6.313255
Cu	2.251573	-1.267686	6.221387
Cu	2.264088	1.261938	6.268781
Cu	2.305320	3.853603	6.368186
Cu	4.458743	-2.560445	6.225651
Cu	4.441388	-0.016282	6.207107
Cu	4.470157	2.580208	6.266850
C	0.696239	3.805629	9.067110
O	0.636895	3.866603	7.635328
H	0.200276	2.891341	9.428871
H	0.194979	4.682670	9.505692
H	1.747505	3.791422	9.397400
H	3.081122	2.477746	7.213240

Initial state of *OH → *O + *H on Cu(111) E= -136.23

Cu	0.000000	0.000000	0.000000
Cu	0.000000	2.571014	0.000000
Cu	0.000000	5.142106	0.000000
Cu	2.226564	-1.285507	0.000000
Cu	2.226564	1.285507	0.000000
Cu	2.226564	3.856599	0.000000
Cu	4.453196	-2.571053	0.000000
Cu	4.453196	-0.000039	0.000000
Cu	4.453196	2.571053	0.000000
Cu	0.742188	1.285507	2.099295
Cu	0.742188	3.856599	2.099295
Cu	0.742188	6.427613	2.099295
Cu	2.968753	0.000000	2.099295
Cu	2.968753	2.571091	2.099295
Cu	2.968753	5.142106	2.099295
Cu	5.195384	-1.285546	2.099295
Cu	5.195384	1.285546	2.099295
Cu	5.195384	3.856560	2.099295
Cu	1.496939	-0.002494	4.195898
Cu	1.492393	2.551197	4.190200
Cu	1.492035	5.153326	4.188094
Cu	3.703639	-1.277315	4.195625
Cu	3.703475	1.272161	4.197221
Cu	3.698574	3.854394	4.168639
Cu	5.938280	-2.569665	4.168639
Cu	5.918264	-0.003720	4.186945
Cu	5.936439	2.563541	4.168744
Cu	-0.025309	-0.005158	6.221626
Cu	-0.000959	2.561994	6.234950
Cu	-0.001958	5.138690	6.234033
Cu	2.179028	-1.375223	6.300402
Cu	2.180104	1.360527	6.304824
Cu	2.228480	3.849876	6.236242
Cu	4.472442	-2.602881	6.220219
Cu	4.548748	-0.004714	6.301618
Cu	4.471065	2.592775	6.222325
O	2.969457	-0.013058	7.581321
H	2.974150	-0.027068	8.554381

Transition state of *OH → *O + *H on Cu(111) E= -134.64

Cu	0.000000	0.000000	0.000000
Cu	0.000000	2.571014	0.000000
Cu	0.000000	5.142106	0.000000
Cu	2.226564	-1.285507	0.000000
Cu	2.226564	1.285507	0.000000
Cu	2.226564	3.856599	0.000000
Cu	4.453196	-2.571053	0.000000
Cu	4.453196	-0.000039	0.000000
Cu	4.453196	2.571053	0.000000
Cu	0.742188	1.285507	2.099295
Cu	0.742188	3.856599	2.099295
Cu	0.742188	6.427613	2.099295
Cu	2.968753	0.000000	2.099295
Cu	2.968753	2.571091	2.099295
Cu	2.968753	5.142106	2.099295
Cu	5.195384	-1.285546	2.099295
Cu	5.195384	1.285546	2.099295
Cu	5.195384	3.856560	2.099295
Cu	1.489235	0.002110	4.195328
Cu	1.492936	2.560067	4.185660
Cu	1.496777	5.169883	4.196510
Cu	3.705732	-1.277806	4.195934
Cu	3.703011	1.278259	4.180176
Cu	3.701546	3.853102	4.177694
Cu	5.935858	-2.568322	4.177220
Cu	5.917243	0.004977	4.184587
Cu	5.932547	2.566308	4.177467
Cu	-0.036406	-0.005834	6.230560
Cu	-0.006787	2.567561	6.225312
Cu	0.008049	5.145540	6.235847
Cu	2.196309	-1.333157	6.353961
Cu	2.148697	1.356877	6.328503

Cu	2.218978	3.867974	6.237559
Cu	4.464447	-2.605829	6.230743
Cu	4.547652	-0.026983	6.327971
Cu	4.446427	2.565568	6.286140
O	2.954463	-0.019919	7.546850
H	3.709091	1.293174	7.216331

Final state of *OH → *O + *H on Cu(111) E= -135.52

Cu	0.000000	0.000000	0.000000
Cu	0.000000	2.571014	0.000000
Cu	0.000000	5.142106	0.000000
Cu	2.226564	-1.285507	0.000000
Cu	2.226564	1.285507	0.000000
Cu	2.226564	3.856599	0.000000
Cu	4.453196	-2.571053	0.000000
Cu	4.453196	-0.000039	0.000000
Cu	4.453196	2.571053	0.000000
Cu	0.742188	1.285507	2.099295
Cu	0.742188	3.856599	2.099295
Cu	0.742188	6.427613	2.099295
Cu	2.968753	0.000000	2.099295
Cu	2.968753	2.571091	2.099295
Cu	2.968753	5.142106	2.099295
Cu	5.195384	-1.285546	2.099295
Cu	5.195384	1.285546	2.099295
Cu	5.195384	3.856560	2.099295
Cu	1.498596	0.007682	4.204908
Cu	1.494137	2.544471	4.181967
Cu	1.496453	5.143665	4.196450
Cu	3.701459	-1.286452	4.191344
Cu	3.708324	1.275352	4.193821
Cu	3.701369	3.857280	4.155583
Cu	5.941542	-2.572105	4.173584
Cu	5.916163	-0.003827	4.185526
Cu	5.920838	2.567979	4.184452
Cu	-0.020826	0.001417	6.227675
Cu	-0.002422	2.563969	6.219885
Cu	-0.014649	5.135579	6.214336
Cu	2.173071	-1.375274	6.332463
Cu	2.196476	1.334781	6.431113
Cu	2.209888	3.856984	6.257215
Cu	4.477000	-2.588365	6.221112
Cu	4.529207	-0.012327	6.330594
Cu	4.463781	2.602680	6.279066
O	2.996886	-0.106297	7.438958
H	3.001247	2.737891	7.205561

Initial state of $*H_2O$ → *OH + *H on $TiO_2(110)$ E= -425.26

Ti	0.000000	0.000000	1.287864
Ti	0.000000	3.298650	4.586367
Ti	-0.001561	-0.000155	7.915604
Ti	-0.025434	3.283036	11.071592
Ti	1.483000	3.298650	1.287864
Ti	1.483000	0.000000	4.586367
Ti	1.486096	3.299510	7.872265
Ti	1.570430	0.024798	11.098453
Ti	2.966000	0.000000	1.287864
Ti	2.966000	3.298650	4.586367
Ti	2.975512	-0.000764	7.805548
Ti	2.982981	3.296209	11.133904
Ti	4.449000	3.298650	1.287864
Ti	4.449000	0.000000	4.586367
Ti	4.451725	3.299604	7.851349
Ti	4.324047	-0.010584	11.348493
O	0.000000	3.298650	0.000000
O	0.000000	0.000000	3.298778
O	0.011057	3.295977	6.654667
O	-0.035458	-0.014836	10.059721
O	0.000000	3.298650	2.575453
O	0.000000	0.000000	5.874231
O	-0.011647	3.302418	9.237781
O	1.483000	1.287793	1.287864
O	1.483000	4.586443	4.586367
O	1.487001	1.299320	7.956610
O	1.443032	4.561076	11.521387
O	1.483000	5.309507	1.287864
O	1.483000	2.010857	4.586367
O	1.487511	5.296870	7.948177
O	1.456346	2.001931	11.459334
O	2.966000	3.298650	0.000000
O	2.966000	0.000000	3.298778
O	2.959586	3.296598	6.657960
O	3.020753	-0.011627	9.932508
O	2.966000	3.298650	2.575453
O	2.966000	0.000000	5.874231
O	2.981853	3.301928	9.289462
O	2.881692	-0.005495	12.496566
O	4.449000	1.287793	1.287864
O	4.449000	4.586443	4.586367
O	4.454172	1.301741	7.923621
O	4.483537	4.574811	11.547067
O	4.449000	5.309507	1.287864
O	4.449000	2.010857	4.586367
O	4.453547	5.294274	7.916472
O	4.482589	1.998858	11.551926
O	0.067311	-0.149633	12.937024
H	0.528401	0.507021	13.492349
H	0.491936	5.565666	13.064768

Transition state of $*H_2O$ → *OH + *H on $TiO_2(110)$ E= -424.92

Ti	0.000000	0.000000	1.287864
Ti	0.000000	3.298650	4.586367
Ti	-0.008569	-0.018341	7.842971
Ti	0.001742	3.278601	11.079417
Ti	1.483000	3.298650	1.287864
Ti	1.483000	0.000000	4.586367
Ti	1.484428	3.279179	7.879497
Ti	1.456649	-0.033925	11.080853
Ti	2.966000	0.000000	1.287864
Ti	2.966000	3.298650	4.586367
Ti	2.970415	-0.015772	7.814490
Ti	2.987336	3.260308	11.119748
Ti	4.449000	3.298650	1.287864
Ti	4.449000	0.000000	4.586367
Ti	4.448793	3.279852	7.847655
Ti	4.273148	-0.044895	11.412191
O	0.000000	3.298650	0.000000
O	0.000000	0.000000	3.298778
O	0.014514	3.284374	6.659083
O	-0.091170	-0.026763	10.110840
O	0.000000	3.298650	2.575453
O	0.000000	0.000000	5.874231
O	-0.022478	3.271924	9.254851
O	1.483000	1.287793	1.287864
O	1.483000	4.586443	4.586367
O	1.478476	1.277359	7.977403
O	1.485218	4.549493	11.390032
O	1.483000	5.309507	1.287864
O	1.483000	2.010857	4.586367
O	1.481157	5.282565	7.976365
O	1.480926	2.001795	11.422389
O	2.966000	3.298650	0.000000
O	2.966000	0.000000	3.298778
O	2.951549	3.282817	6.660680
O	3.014680	-0.009724	10.015917
O	2.966000	3.298650	2.575453
O	2.966000	0.000000	5.874231
O	2.996231	3.266894	9.285307
O	2.898057	-0.066982	12.645990
O	4.449000	1.287793	1.287864
O	4.449000	4.586443	4.586367
O	4.448990	1.280424	7.924781
O	4.473826	4.546944	11.512672
O	4.449000	5.309507	1.287864
O	4.449000	2.010857	4.586367
O	4.446087	5.279979	7.923442
O	4.490405	1.983195	11.560414
O	0.532182	-0.012013	13.022599
H	0.351672	0.912490	13.286353
H	1.735792	-0.092951	13.148510

Final state of $*H_2O$ → *OH + *H on $TiO_2(110)$ E= -426.02

Ti	0.000000	0.000000	1.287864
Ti	0.000000	3.298650	4.586367
Ti	-0.001911	-0.025294	7.863886
Ti	-0.000808	3.287847	11.085659
Ti	1.483000	3.298650	1.287864
Ti	1.483000	0.000000	4.586367
Ti	1.480885	3.276656	7.850118
Ti	1.479541	-0.041788	11.257892
Ti	2.966000	0.000000	1.287864
Ti	2.966000	3.298650	4.586367
Ti	2.964185	-0.017357	7.865485
Ti	2.959538	3.229094	11.084843
Ti	4.449000	3.298650	1.287864
Ti	4.449000	0.000000	4.586367
Ti	4.446725	3.276551	7.855608
Ti	4.445807	-0.041459	11.253300
O	0.000000	3.298650	0.000000
O	0.000000	0.000000	3.298778
O	-0.003911	3.278836	6.649184
O	-0.003058	-0.071074	10.011769
O	0.000000	3.298650	2.575453
O	0.000000	0.000000	5.874231
O	0.000254	3.284981	9.255026
O	1.483000	1.287793	1.287864
O	1.483000	4.586443	4.586367
O	1.476778	1.277867	7.943365
O	1.496306	4.532996	11.483240
O	1.483000	5.309507	1.287864
O	1.483000	2.010857	4.586367
O	1.485346	5.276040	7.927185
O	1.462481	1.978100	11.473437
O	2.966000	3.298650	0.000000
O	2.966000	0.000000	3.298778
O	2.965932	3.277033	6.649099
O	2.962551	-0.013907	10.011642
O	2.966000	3.298650	2.575453
O	2.966000	0.000000	5.874231
O	2.960550	3.267998	9.254132
O	2.964277	-0.036088	12.678679
O	4.449000	1.287793	1.287864
O	4.449000	4.586443	4.586367
O	4.451316	1.278068	7.945173
O	4.428463	4.530965	11.476074
O	4.449000	5.309507	1.287864
O	4.449000	2.010857	4.586367
O	4.442871	5.275842	7.929056
O	4.461963	1.979952	11.466523
O	-0.005572	-0.053909	12.678788
H	-0.007900	0.732787	13.251527
H	2.966524	5.763198	13.235889

Initial state of $*CH_3OH$ → $*CH_3O$ + *H on $TiO_2(110)$ E= -441.34

Ti	0.000000	0.000000	1.287864
Ti	0.000000	3.298650	4.586367
Ti	-0.006263	0.003944	7.914906
Ti	-0.018882	3.316258	11.063224
Ti	1.483000	3.298650	1.287864
Ti	1.483000	0.000000	4.586367
Ti	1.479427	3.301105	7.862993
Ti	1.576090	-0.032275	11.206732
Ti	2.966000	0.000000	1.287864
Ti	2.966000	3.298650	4.586367
Ti	2.962051	0.002614	7.810638
Ti	2.962774	3.291426	11.133216
Ti	4.449000	3.298650	1.287864
Ti	4.449000	0.000000	4.586367
Ti	4.443675	3.301165	7.855385
Ti	4.330444	-0.000617	11.270729
O	0.000000	3.298650	0.000000
O	0.000000	0.000000	3.298778
O	-0.001660	3.301868	6.652200
O	-0.013596	0.013142	10.079559
O	0.000000	3.298650	2.575453
O	0.000000	0.000000	5.874231
O	-0.009767	3.297073	9.236502
O	1.483000	1.287793	1.287864
O	1.483000	4.586443	4.586367
O	1.477360	1.301546	7.938800
O	1.450223	4.589533	11.489523
O	1.483000	5.309507	1.287864
O	1.483000	2.010857	4.586367
O	1.478170	5.299027	7.947684
O	1.427282	2.034895	11.546897
O	2.966000	3.298650	0.000000
O	2.966000	0.000000	3.298778
O	2.958751	3.301910	6.655389
O	2.970495	0.014573	9.941876
O	2.966000	3.298650	2.575453
O	2.966000	0.000000	5.874231
O	2.963540	3.290519	9.292259
O	2.940350	0.007365	12.516549
O	4.449000	1.287793	1.287864
O	4.449000	4.586443	4.586367
O	4.445381	1.301993	7.931886
O	4.468638	4.592257	11.522759
O	4.449000	5.309507	1.287864
O	4.449000	2.010857	4.586367
O	4.444720	5.298838	7.940478
O	4.476060	2.031237	11.531788
O	0.015185	0.159814	12.903613
C	0.053488	6.052180	14.163947
H	5.150700	6.388627	14.794599
H	-0.068252	4.991609	13.924141
H	1.027036	6.220558	14.644270
H	0.174939	1.124716	13.016345

Transition state of $*CH_3OH$ → $*CH_3O$ + *H on $TiO_2(110)$ E= -440.90

Ti	0.000000	0.000000	1.287864
Ti	0.000000	3.298650	4.586367
Ti	-0.019192	0.031285	7.817068
Ti	-0.014536	3.341960	11.087649
Ti	1.483000	3.298650	1.287864
Ti	1.483000	0.000000	4.586367
Ti	1.477559	3.330908	7.881327
Ti	1.413077	0.029298	11.209382
Ti	2.966000	0.000000	1.287864
Ti	2.966000	3.298650	4.586367
Ti	2.951122	0.032836	7.808707
Ti	2.973968	3.356770	11.121539
Ti	4.449000	3.298650	1.287864
Ti	4.449000	0.000000	4.586367
Ti	4.440464	3.333775	7.861119
Ti	4.235199	0.063189	11.341425
O	0.000000	3.298650	0.000000
O	0.000000	0.000000	3.298778
O	-0.001135	3.325556	6.660657
O	-0.074166	0.054948	10.162982
O	0.000000	3.298650	2.575453
O	0.000000	0.000000	5.874231
O	-0.021021	3.339466	9.254066
O	1.483000	1.287793	1.287864
O	1.483000	4.586443	4.586367
O	1.465634	1.327388	7.954186
O	1.469072	4.631848	11.465285
O	1.483000	5.309507	1.287864
O	1.483000	2.010857	4.586367
O	1.463728	5.334485	7.965782
O	1.484005	2.085361	11.423926
O	2.966000	3.298650	0.000000
O	2.966000	0.000000	3.298778
O	2.951043	3.327019	6.661967
O	2.919204	0.034957	10.035039
O	2.966000	3.298650	2.575453
O	2.966000	0.000000	5.874231
O	2.972333	3.343312	9.276318
O	3.001330	0.100890	12.697929
O	4.449000	1.287793	1.287864
O	4.449000	4.586443	4.586367
O	4.434392	1.327081	7.942174
O	4.467170	4.647523	11.516678
O	4.449000	5.309507	1.287864
O	4.449000	2.010857	4.586367
O	4.436942	5.334790	7.950924
O	4.462011	2.074504	11.499909
O	0.682112	0.161119	13.181074
C	0.209689	5.824850	14.159597
H	5.186951	6.196629	14.562403
H	0.041714	4.826624	13.726481
H	0.941159	5.740039	14.978456
H	1.902261	0.201563	13.227284

Final state of $*CH_3OH$ → $*CH_3O$ + *H on $TiO_2(110)$ E= -442.09

Ti	0.000000	0.000000	1.287864
Ti	0.000000	3.298650	4.586367
Ti	-0.001576	-0.003074	7.857100
Ti	-0.000954	3.257196	11.089062
Ti	1.483000	3.298650	1.287864
Ti	1.483000	0.000000	4.586367
Ti	1.481114	3.285758	7.849646
Ti	1.475483	-0.036626	11.258171
Ti	2.966000	0.000000	1.287864
Ti	2.966000	3.298650	4.586367
Ti	2.964054	-0.019777	7.866546
Ti	2.961709	3.300116	11.073440
Ti	4.449000	3.298650	1.287864
Ti	4.449000	0.000000	4.586367
Ti	4.448038	3.285849	7.852132
Ti	4.447807	-0.035971	11.252976
O	0.000000	3.298650	0.000000
O	0.000000	0.000000	3.298778
O	-0.002277	3.282614	6.646760
O	-0.003893	0.026343	10.019946
O	0.000000	3.298650	2.575453
O	0.000000	0.000000	5.874231
O	-0.000550	3.275044	9.257060

O	1.483000	1.287793	1.287864
O	1.483000	4.586443	4.586367
O	1.486253	1.286637	7.936331
O	1.477099	4.567042	11.461524
O	1.483000	5.309507	1.287864
O	1.483000	2.010857	4.586367
O	1.474166	5.284012	7.935795
O	1.496258	2.016529	11.485408
O	2.966000	3.298650	0.000000
O	2.966000	0.000000	3.298778
O	2.965480	3.290929	6.646824
O	2.960602	-0.038888	10.014078
O	2.966000	3.298650	2.575453
O	2.966000	0.000000	5.874231
O	2.962850	3.284636	9.245962
O	2.961925	0.015221	12.697467
O	4.449000	1.287793	1.287864
O	4.449000	4.586643	4.586367
O	4.441935	1.286661	7.937262
O	4.448783	4.567683	11.457314
O	4.449000	5.309507	1.287864
O	4.449000	2.010857	4.586367
O	4.453959	5.283905	7.936749
O	4.429717	2.018329	11.483248
O	-0.006766	-0.039855	12.660034
C	-0.021337	5.943837	13.932348
H	5.078749	6.348776	14.531694
H	5.765475	4.852113	13.839377
H	0.935418	6.127981	14.446958
H	2.963313	0.891581	13.124746

Initial state of *OH → *O + *H on TiO_2(110) E= -422.52

Ti	0.000000	0.000000	1.287864
Ti	0.000000	3.298650	4.586367
Ti	0.010658	-0.018776	8.017486
Ti	0.014437	3.296448	11.061543
Ti	1.483000	3.298650	1.287864
Ti	1.483000	0.000000	4.586367
Ti	1.492644	3.282530	7.843189
Ti	1.569070	-0.023418	11.313191
Ti	2.966000	0.000000	1.287864
Ti	2.966000	3.298650	4.586367
Ti	2.976427	-0.009977	7.859945
Ti	2.980334	3.271359	11.074108
Ti	4.449000	3.298650	1.287864
Ti	4.449000	0.000000	4.586367
Ti	4.458014	3.282594	7.842536
Ti	4.395261	-0.023339	11.306046
O	0.000000	3.298650	0.000000
O	0.000000	0.000000	3.298778
O	0.008727	3.288349	6.643662
O	0.012344	-0.025748	9.983988
O	0.000000	3.298650	2.575453
O	0.000000	0.000000	5.874231
O	0.010518	3.280330	9.233789
O	1.483000	1.287793	1.287864
O	1.483000	4.586443	4.586367
O	1.491730	1.286606	7.931390
O	1.499258	4.547348	11.407319
O	1.483000	5.309507	1.287864
O	1.483000	2.010857	4.586367
O	1.499797	5.279647	7.931289
O	1.485627	2.033627	11.444385
O	2.966000	3.298650	0.000000
O	2.966000	0.000000	3.298778
O	2.974281	3.283391	6.642338
O	2.979190	0.005604	9.966419
O	2.966000	3.298650	2.575453
O	2.966000	0.000000	5.874231
O	2.976664	3.272123	9.243980
O	2.981817	-0.006737	12.514387
O	4.449000	1.287793	1.287864
O	4.449000	4.586443	4.586367
O	4.458382	1.286781	7.930156
O	4.462329	4.547387	11.406266
O	4.449000	5.309507	1.287864
O	4.449000	2.010857	4.586367
O	4.450320	5.279657	7.929949
O	4.476069	2.033487	11.443280
O	0.014257	-0.032997	12.625271
H	0.012052	0.615399	13.349818

Transition state of *OH → *O + *H on TiO_2(110) E= -421.27

Ti	0.000000	0.000000	1.287864
Ti	0.000000	3.298650	4.586367
Ti	-0.019113	0.004362	7.908409
Ti	-0.001534	3.306612	11.059999
Ti	1.483000	3.298650	1.287864
Ti	1.483000	0.000000	4.586367
Ti	1.481657	3.303258	7.865072
Ti	1.616941	0.007968	10.986146
Ti	2.966000	0.000000	1.287864
Ti	2.966000	3.298650	4.586367
Ti	2.975643	0.004405	7.895249
Ti	2.970603	3.306535	11.061704
Ti	4.449000	3.298650	1.287864
Ti	4.449000	0.000000	4.586367
Ti	4.448584	3.303034	7.819579
Ti	4.613839	0.008293	11.572712
O	0.000000	3.298650	0.000000
O	0.000000	0.000000	3.298778
O	0.022009	3.302978	6.639760
O	-0.110434	0.008226	10.049565
O	0.000000	3.298650	2.575453
O	0.000000	0.000000	5.874231
O	-0.041894	3.303416	9.230545
O	1.483000	1.287793	1.287864
O	1.483000	4.586443	4.586367
O	1.474447	1.304967	7.975660
O	1.482965	4.548129	11.285992
O	1.483000	5.309507	1.287864
O	1.483000	2.010857	4.586367
O	1.474576	5.301320	7.978316
O	1.482959	2.066090	11.288366
O	2.966000	3.298650	0.000000
O	2.966000	0.000000	3.298778
O	2.942168	3.302897	6.641064
O	3.098052	0.008141	9.926781
O	2.966000	3.298650	2.575453
O	2.966000	0.000000	5.874231
O	3.005227	3.303334	9.230210
O	2.653437	0.008295	12.558777
O	4.449000	1.287793	1.287864
O	4.449000	4.586443	4.586367
O	4.453117	1.309732	7.881271
O	4.449266	4.568031	11.489104
O	4.449000	5.309507	1.287864
O	4.449000	2.010857	4.586367
O	4.453030	5.296452	7.884553
O	4.449274	2.045893	11.490879
O	0.180730	0.008340	12.651242
H	1.414438	0.008706	12.961523

Final state of *OH → *O + *H on TiO_2(110) E= -422.52

Ti	0.000000	0.000000	1.287864
Ti	0.000000	3.298650	4.586367
Ti	0.008238	-0.010957	7.860096
Ti	0.012689	3.277369	11.072145
Ti	1.483000	3.298650	1.287864
Ti	1.483000	0.000000	4.586367
Ti	1.490524	3.284970	7.842330
Ti	1.426893	-0.022731	11.307044
Ti	2.966000	0.000000	1.287864
Ti	2.966000	3.298650	4.586367
Ti	2.974214	-0.013104	8.013732
Ti	2.978698	3.291369	11.059171
Ti	4.449000	3.298650	1.287864
Ti	4.449000	0.000000	4.586367
Ti	4.457178	3.284986	7.842197
Ti	4.532677	-0.023014	11.305827
O	0.000000	3.298650	0.000000
O	0.000000	0.000000	3.298778
O	0.007292	3.287902	6.641624
O	0.012432	0.003682	9.962839
O	0.000000	3.298650	2.575453
O	0.000000	0.000000	5.874231
O	0.009357	3.276762	9.242239
O	1.483000	1.287793	1.287864
O	1.483000	4.586443	4.586367
O	1.488681	1.289336	7.927419
O	1.498258	4.548968	11.404793
O	1.483000	5.309507	1.287864
O	1.483000	2.010857	4.586367
O	1.485650	5.281283	7.929247
O	1.505063	2.034311	11.442823
O	2.966000	3.298650	0.000000
O	2.966000	0.000000	3.298778
O	2.973312	3.288623	6.642711
O	2.977227	-0.017963	9.980892
O	2.966000	3.298650	2.575453
O	2.966000	0.000000	5.874231
O	2.975153	3.277252	9.231694
O	2.979702	-0.026997	12.620566
O	4.449000	1.287793	1.287864
O	4.449000	4.586443	4.586367
O	4.458989	1.289272	7.927602
O	4.460083	4.548868	11.404099
O	4.449000	5.309507	1.287864
O	4.449000	2.010857	4.586367
O	4.461954	5.281236	7.929488
O	4.453356	2.034361	11.442293
O	0.012829	-0.008804	12.511548
H	2.981864	0.616379	13.350221

Initial state of *H_2O → *OH + *H on Cu/ZnO E= -414.95

O	12.869584	2.866686	6.301443
O	8.601952	0.484005	6.598915
O	5.160166	6.446134	6.751906
O	6.885656	3.456537	6.805943
O	1.724872	6.436317	6.870178
O	5.159389	0.485244	6.834499
O	3.440710	3.463759	6.802449
O	10.173312	3.277625	6.270559
O	11.850865	0.365712	6.273743
O	8.212593	6.139437	5.844183
Zn	3.430419	7.392317	6.621651
Zn	0.012010	7.426368	6.749237
Zn	1.743275	4.461753	6.673922
Zn	5.166441	4.442432	6.559779
Zn	6.849205	1.490283	6.591272
Zn	3.475735	1.499957	6.581997
Zn	8.540363	4.269649	6.428121
Zn	6.769960	7.373428	6.419597
Zn	10.242037	1.374904	6.494950
Cu	-0.006544	2.791872	4.235274
Cu	6.389698	4.975031	4.148501
Cu	5.109059	2.756850	4.171724
Cu	2.475006	7.175027	4.151562
Cu	1.295220	0.551422	4.240363
Cu	7.642709	2.748762	4.149463
Cu	1.234605	4.964200	4.193139
Cu	3.796845	0.523534	4.212953
Cu	-1.300845	5.009000	4.237786
Cu	8.878182	0.481992	4.265605
Cu	-0.070811	7.195829	4.171416
Cu	6.333094	0.507384	4.154470
Cu	3.809724	4.971790	4.163651
Cu	-2.576878	7.221282	4.234641
Cu	5.062492	7.167485	4.185779
Cu	2.528495	2.755798	4.311384
Cu	-3.860813	7.969264	2.101587
Cu	0.000000	1.282144	2.101587
Cu	2.573875	1.282144	2.101587
Cu	5.147750	1.282144	2.101587
Cu	7.721625	1.282144	2.101587
Cu	-1.286937	3.511184	2.101587
Cu	-2.573875	5.740224	2.101587
Cu	1.286938	3.511184	2.101587
Cu	6.434688	3.511184	2.101587
Cu	3.860813	3.511184	2.101587
Cu	0.000000	5.740224	2.101587
Cu	2.573875	5.740224	2.101587
Cu	5.147750	5.740224	2.101587
Cu	-1.286937	7.969264	2.101587
Cu	1.286938	7.969264	2.101587
Cu	3.860813	7.969264	2.101587
Cu	2.573926	4.254167	0.000000
Cu	2.573926	8.712247	0.000000
Cu	6.434739	6.483207	0.000000
Cu	5.147801	8.712247	0.000000
Cu	1.286989	6.483207	0.000000
Cu	-2.573824	8.712247	0.000000
Cu	1.286989	2.025127	0.000000
Cu	9.008614	2.025127	0.000000
Cu	0.000051	8.712247	0.000000
Cu	-1.286886	6.483207	0.000000
Cu	6.434739	2.025127	0.000000
Cu	7.721676	4.254167	0.000000
Cu	0.000051	4.254167	0.000000
Cu	3.860864	2.025127	0.000000
Cu	5.147801	4.254167	0.000000
Cu	3.860864	6.483207	0.000000
Cu	10.241301	2.855869	4.231694
Cu	16.741167	5.017650	4.162753
Cu	15.502092	2.795720	4.159257
Cu	12.904497	7.276727	4.195347
Cu	11.588584	0.497130	4.226733
Cu	18.043847	2.787698	4.175440
Cu	11.625397	5.095978	4.135266
Cu	14.229191	0.569844	4.138494
Cu	9.035574	5.076299	4.099937
Cu	19.324964	0.567133	4.172325
Cu	10.369093	7.294944	4.186319
Cu	16.762056	0.579109	4.198038
Cu	14.197569	5.071642	4.157656
Cu	7.768322	7.226783	4.108117
Cu	15.456930	7.266670	4.170037
Cu	12.936405	2.842133	4.204546
Cu	6.434688	7.969264	2.101587
Cu	10.295500	1.282144	2.101587
Cu	12.869375	1.282144	2.101587
Cu	15.443250	1.282144	2.101587
Cu	18.017125	1.282144	2.101587
Cu	9.008563	3.511184	2.101587
Cu	7.721625	5.740224	2.101587
Cu	11.582438	3.511184	2.101587
Cu	16.730188	3.511184	2.101587
Cu	14.156313	3.511184	2.101587
Cu	10.295500	5.740224	2.101587
Cu	12.869375	5.740224	2.101587
Cu	15.443250	5.740224	2.101587
Cu	9.008563	7.969264	2.101587
Cu	11.582438	7.969264	2.101587
Cu	14.156313	7.969264	2.101587
Cu	12.869426	4.254167	0.000000
Cu	12.869426	8.712247	0.000000
Cu	-3.860761	6.483207	0.000000
Cu	-5.147699	8.712247	0.000000
Cu	11.582489	6.483207	0.000000
Cu	7.721676	8.712247	0.000000
Cu	11.582489	2.025127	0.000000
Cu	-1.286886	2.025127	0.000000
Cu	10.295551	8.712247	0.000000
Cu	9.008614	6.483207	0.000000
Cu	16.730239	2.025127	0.000000
Cu	-2.573824	4.254167	0.000000
Cu	10.295551	4.254167	0.000000
Cu	14.156364	2.025127	0.000000
Cu	15.443301	4.254167	0.000000
Cu	14.156364	6.483207	0.000000
H	12.724365	1.882277	6.461014
H	11.958726	3.264371	6.458299

Transition state of *H_2O → *OH + *H on Cu/ZnO E= -414.82

O	13.375921	2.228534	6.105689
O	8.601952	0.484005	6.598915
O	5.160166	6.446134	6.751906
O	6.885656	3.456537	6.805943
O	1.724872	6.436317	6.870178
O	5.159389	0.485244	6.834499
O	3.440710	3.463759	6.802449
O	10.096113	3.324700	6.188283
O	11.803973	0.399094	6.384576
O	8.244365	6.210580	5.887065
Zn	3.430419	7.392317	6.621651
Zn	0.012010	7.426368	6.749237
Zn	1.743275	4.461753	6.673922
Zn	5.166441	4.442432	6.559779
Zn	6.849205	1.490283	6.591272
Zn	3.475735	1.499957	6.581997
Zn	8.500226	4.339315	6.428129
Zn	6.765504	7.389335	6.436848
Zn	10.209124	1.462649	6.508836
Cu	-0.013173	2.786601	4.233650
Cu	6.390480	4.966648	4.139622
Cu	5.105614	2.752308	4.169917
Cu	2.468943	7.163430	4.153945
Cu	1.288249	0.541159	4.242549
Cu	7.641880	2.745748	4.153619
Cu	1.233280	4.955603	4.192926
Cu	3.787621	0.523777	4.209257
Cu	-1.304080	5.007447	4.237971
Cu	8.870094	0.464397	4.276843
Cu	-0.075636	7.193070	4.170506
Cu	6.327415	0.501791	4.158021
Cu	3.807556	4.963518	4.162535
Cu	-2.578428	7.215906	4.235628
Cu	5.061675	7.154199	4.195008
Cu	2.523697	2.749359	4.313716
Cu	-3.860812	7.969264	2.101587
Cu	-0.000000	1.282144	2.101587
Cu	2.573875	1.282144	2.101587
Cu	5.147750	1.282144	2.101587

Cu	7.721625	1.282144	2.101587
Cu	-1.286938	3.511184	2.101587
Cu	-2.573875	5.740224	2.101587
Cu	1.286938	3.511184	2.101587
Cu	6.434688	3.511184	2.101587
Cu	3.860812	3.511184	2.101587
Cu	-0.000000	5.740224	2.101587
Cu	2.573875	5.740224	2.101587
Cu	5.147750	5.740224	2.101587
Cu	-1.286937	7.969264	2.101587
Cu	1.286937	7.969264	2.101587
Cu	3.860813	7.969264	2.101587
Cu	2.573926	4.254167	0.000000
Cu	2.573926	8.712247	0.000000
Cu	6.434739	6.483207	0.000000
Cu	5.147801	8.712247	0.000000
Cu	1.286989	6.483207	0.000000
Cu	-2.573824	8.712247	0.000000
Cu	1.286989	2.025127	0.000000
Cu	9.008614	2.025127	0.000000
Cu	0.000051	8.712247	0.000000
Cu	-1.286886	6.483207	0.000000
Cu	6.434739	2.025127	0.000000
Cu	7.721676	4.254167	0.000000
Cu	0.000051	4.254167	0.000000
Cu	3.860864	2.025127	0.000000
Cu	5.147801	4.254167	0.000000
Cu	3.860864	6.483207	0.000000
Cu	10.259418	2.848269	4.225148
Cu	16.734330	5.014863	4.165721
Cu	15.508682	2.795664	4.159283
Cu	12.906211	7.261686	4.198508
Cu	11.533028	0.471674	4.210141
Cu	18.046888	2.781605	4.175250
Cu	11.639782	5.072757	4.161959
Cu	14.235679	0.531834	4.115816
Cu	9.038215	5.071739	4.090952
Cu	19.321345	0.561005	4.171699
Cu	10.372411	7.273958	4.192617
Cu	16.761969	0.568151	4.191734
Cu	14.193459	5.054911	4.158910
Cu	7.754397	7.221596	4.117961
Cu	15.453807	7.259512	4.170542
Cu	12.902098	2.808812	4.199783
Cu	6.434687	7.969264	2.101587
Cu	10.295500	1.282144	2.101587
Cu	12.869375	1.282144	2.101587
Cu	15.443250	1.282144	2.101587
Cu	18.017125	1.282144	2.101587
Cu	9.008562	3.511184	2.101587
Cu	7.721625	5.740224	2.101587
Cu	11.582438	3.511184	2.101587
Cu	16.730187	3.511184	2.101587
Cu	14.156313	3.511184	2.101587
Cu	10.295500	5.740224	2.101587
Cu	12.869375	5.740224	2.101587
Cu	15.443250	5.740224	2.101587
Cu	9.008562	7.969264	2.101587
Cu	11.582438	7.969264	2.101587
Cu	14.156312	7.969264	2.101587
Cu	12.869426	4.254167	0.000000
Cu	12.869426	8.712247	0.000000
Cu	-3.860761	6.483207	0.000000
Cu	-5.147699	8.712247	0.000000
Cu	11.582489	6.483207	0.000000
Cu	7.721676	8.712247	0.000000
Cu	11.582489	2.025127	0.000000
Cu	-1.286886	2.025127	0.000000
Cu	10.295551	8.712247	0.000000
Cu	9.008614	6.483207	0.000000
Cu	16.730239	2.025127	0.000000
Cu	-2.573824	4.254167	0.000000
Cu	10.295551	4.254167	0.000000
Cu	14.156364	2.025127	0.000000
Cu	15.443301	4.254167	0.000000
Cu	14.156364	6.483207	0.000000
H	12.693682	1.330615	6.348536
H	13.237540	2.956545	6.739125

Final state of $*H_2O \rightarrow *OH + *H$ on Cu/ZnO E= -415.19

O	12.894038	4.260167	5.501218
O	8.601952	0.484005	6.598915
O	5.160166	6.446134	6.751906
O	6.885656	3.456537	6.805943
O	1.724872	6.436317	6.870178
O	5.159389	0.485244	6.834499
O	3.440710	3.463759	6.802449
O	10.047110	3.346533	7.070033
O	11.782371	0.404873	6.262396
O	8.221702	6.257184	5.929135
Zn	3.430419	7.392317	6.621651
Zn	0.012010	7.426368	6.749237
Zn	1.743275	4.461753	6.673922
Zn	5.166441	4.442432	6.559779
Zn	6.849205	1.490283	6.591272
Zn	3.475735	1.499957	6.581997
Zn	8.474810	4.399608	6.478034
Zn	6.744298	7.438696	6.450455
Zn	10.243804	1.461065	6.569745
Cu	-0.001063	2.792784	4.239206
Cu	6.387238	4.968204	4.141811
Cu	5.116844	2.749673	4.169407
Cu	2.489197	7.189691	4.138989
Cu	1.297381	0.552197	4.240915
Cu	7.672386	2.768395	4.183963
Cu	1.243212	4.966121	4.193356
Cu	3.799612	0.521983	4.213821
Cu	-1.294934	5.008581	4.240870
Cu	8.890843	0.504325	4.319423
Cu	-0.062953	7.199816	4.171049
Cu	6.341573	0.514459	4.150234
Cu	3.811734	4.969825	4.157709
Cu	-2.566774	7.218602	4.243792
Cu	5.070591	7.167383	4.188425
Cu	2.532819	2.757405	4.304384

Cu	-3.860812	7.969264	2.101587
Cu	-0.000000	1.282144	2.101587
Cu	2.573875	1.282144	2.101587
Cu	5.147750	1.282144	2.101587
Cu	7.721625	1.282144	2.101587
Cu	-1.286937	3.511184	2.101587
Cu	-2.573875	5.740224	2.101587
Cu	1.286938	3.511184	2.101587
Cu	6.434688	3.511184	2.101587
Cu	3.860812	3.511184	2.101587
Cu	0.000000	5.740224	2.101587
Cu	2.573875	5.740224	2.101587
Cu	5.147750	5.740224	2.101587
Cu	-1.286937	7.969264	2.101587
Cu	1.286937	7.969264	2.101587
Cu	3.860813	7.969264	2.101587
Cu	2.573926	4.254167	0.000000
Cu	2.573926	8.712247	0.000000
Cu	6.434739	6.483207	0.000000
Cu	5.147801	8.712247	0.000000
Cu	1.286989	6.483207	0.000000
Cu	-2.573824	8.712247	0.000000
Cu	1.286989	2.025127	0.000000
Cu	9.008614	2.025127	0.000000
Cu	0.000051	8.712247	0.000000
Cu	-1.286886	6.483207	0.000000
Cu	6.434739	2.025127	0.000000
Cu	7.721676	4.254167	0.000000
Cu	0.000051	4.254167	0.000000
Cu	3.860864	2.025127	0.000000
Cu	5.147801	4.254167	0.000000
Cu	3.860864	6.483207	0.000000
Cu	10.224178	2.764156	4.121599
Cu	16.762883	5.011871	4.158026
Cu	15.502706	2.767683	4.154746
Cu	12.897092	7.306793	4.155232
Cu	11.572687	0.507141	4.264054
Cu	18.042493	2.785265	4.174018
Cu	11.528002	5.077957	4.195025
Cu	14.226003	0.546546	4.154634
Cu	9.032921	4.994058	4.106398
Cu	19.331802	0.575516	4.181579
Cu	10.349534	7.279242	4.174248
Cu	16.753751	0.569795	4.201468
Cu	14.255616	5.062461	4.187049
Cu	7.774941	7.188968	4.134498
Cu	15.465314	7.273978	4.161063
Cu	12.896649	2.686040	4.188815
Cu	6.434687	7.969264	2.101587
Cu	10.295500	1.282144	2.101587
Cu	12.869375	1.282144	2.101587
Cu	15.443250	1.282144	2.101587
Cu	18.017125	1.282144	2.101587
Cu	9.008562	3.511184	2.101587
Cu	7.721625	5.740224	2.101587
Cu	11.582438	3.511184	2.101587
Cu	16.730188	3.511184	2.101587
Cu	14.156313	3.511184	2.101587
Cu	10.295500	5.740224	2.101587
Cu	12.869375	5.740224	2.101587
Cu	15.443250	5.740224	2.101587
Cu	9.008563	7.969264	2.101587
Cu	11.582438	7.969264	2.101587
Cu	14.156312	7.969264	2.101587
Cu	12.869426	4.254167	0.000000
Cu	12.869426	8.712247	0.000000
Cu	-3.860761	6.483207	0.000000
Cu	-5.147699	8.712247	0.000000
Cu	11.582489	6.483207	0.000000
Cu	7.721676	8.712247	0.000000
Cu	11.582489	2.025127	0.000000
Cu	-1.286886	2.025127	0.000000
Cu	10.295551	8.712247	0.000000
Cu	9.008614	6.483207	0.000000
Cu	16.730239	2.025127	0.000000
Cu	-2.573824	4.254167	0.000000
Cu	10.295551	4.254167	0.000000
Cu	14.156364	2.025127	0.000000
Cu	15.443301	4.254167	0.000000
Cu	14.156364	6.483207	0.000000
H	10.858177	3.820597	6.795693
H	13.150079	4.325400	6.440303

Initial state of $*CH_3OH \rightarrow *CH_3O + *H$ on Cu/ZnO E= -430.80

O	11.601458	5.169127	6.276503
O	8.601952	0.484005	6.598915
O	5.160166	6.446134	6.751906
O	6.885656	3.456537	6.805943
O	1.724872	6.436317	6.870178
O	5.159389	0.485244	6.834499
O	3.440710	3.463759	6.802449
O	10.103657	3.238398	6.360342
O	11.831788	0.310352	6.229701
O	8.257631	6.157724	5.845349
Zn	3.430419	7.392317	6.621651
Zn	0.012010	7.426368	6.749237
Zn	1.743275	4.461753	6.673922
Zn	5.166441	4.442432	6.559779
Zn	6.849205	1.490283	6.591272
Zn	3.475735	1.499957	6.581997
Zn	8.501706	4.294002	6.422206
Zn	6.784633	7.343239	6.417663
Zn	10.295743	1.356232	6.542267
Cu	-0.010214	2.791667	4.240766
Cu	6.381762	4.974422	4.137226
Cu	5.107264	2.754961	4.175144
Cu	2.480630	7.175743	4.148879
Cu	1.292225	0.550692	4.242633
Cu	7.647297	2.750290	4.149273
Cu	1.233948	4.962072	4.190925
Cu	3.798947	0.523372	4.212908
Cu	-1.297748	5.009601	4.233790
Cu	8.874311	0.484638	4.287081
Cu	-0.065891	7.199360	4.169997
Cu	6.332445	0.509167	4.154886

Cu	3.811117	4.972103	4.160263
Cu	-2.574423	7.226299	4.236750
Cu	5.068354	7.176879	4.184386
Cu	2.529329	2.756725	4.300630
Cu	-3.860813	7.969264	2.101587
Cu	-0.000000	1.282144	2.101587
Cu	2.573875	1.282144	2.101587
Cu	5.147750	1.282144	2.101587
Cu	7.721625	1.282144	2.101587
Cu	-1.286937	3.511184	2.101587
Cu	-2.573875	5.740224	2.101587
Cu	1.286938	3.511184	2.101587
Cu	6.434688	3.511184	2.101587
Cu	3.860812	3.511184	2.101587
Cu	-0.000000	5.740224	2.101587
Cu	2.573875	5.740224	2.101587
Cu	5.147750	5.740224	2.101587
Cu	-1.286937	7.969264	2.101587
Cu	1.286937	7.969264	2.101587
Cu	3.860812	7.969264	2.101587
Cu	2.573926	4.254167	0.000000
Cu	2.573926	8.712247	0.000000
Cu	6.434739	6.483207	0.000000
Cu	5.147801	8.712247	0.000000
Cu	1.286989	6.483207	0.000000
Cu	-2.573824	8.712247	0.000000
Cu	1.286989	2.025127	0.000000
Cu	9.008614	2.025127	0.000000
Cu	0.000051	8.712247	0.000000
Cu	-1.286886	6.483207	0.000000
Cu	6.434739	2.025127	0.000000
Cu	7.721676	4.254167	0.000000
Cu	0.000051	4.254167	0.000000
Cu	3.860864	2.025127	0.000000
Cu	5.147801	4.254167	0.000000
Cu	3.860864	6.483207	0.000000
Cu	10.256472	2.838740	4.242049
Cu	16.751906	5.017589	4.167544
Cu	15.463469	2.804033	4.187890
Cu	12.928727	7.315540	4.150249
Cu	11.608582	0.527812	4.245775
Cu	18.029052	2.796044	4.173542
Cu	11.672073	5.099569	4.218774
Cu	14.210946	0.596736	4.174773
H	9.017103	5.060646	4.087914
Cu	19.325820	0.585707	4.177656
Cu	10.358123	7.321977	4.166910
Cu	16.759876	0.591312	4.196634
Cu	14.226536	5.024058	4.146750
Cu	7.771292	7.226726	4.101481
Cu	15.458148	7.259259	4.165565
Cu	12.910209	2.800268	4.127645
Cu	6.434688	7.969264	2.101587
Cu	10.295500	1.282144	2.101587
Cu	12.869375	1.282144	2.101587
Cu	15.443250	1.282144	2.101587
Cu	18.017125	1.282144	2.101587
Cu	9.008563	3.511184	2.101587
Cu	7.721625	5.740224	2.101587
Cu	11.582438	3.511184	2.101587
Cu	16.730188	3.511184	2.101587
Cu	14.156313	3.511184	2.101587
Cu	10.295500	5.740224	2.101587
Cu	12.869375	5.740224	2.101587
Cu	15.443250	5.740224	2.101587
Cu	9.008563	7.969264	2.101587
Cu	11.582438	7.969264	2.101587
Cu	14.156313	7.969264	2.101587
Cu	12.869426	4.254167	0.000000
Cu	12.869426	8.712247	0.000000
Cu	-3.860761	6.483207	0.000000
Cu	-5.147699	8.712247	0.000000
Cu	11.582489	6.483207	0.000000
Cu	7.721676	8.712247	0.000000
Cu	11.582489	2.025127	0.000000
Cu	-1.286886	2.025127	0.000000
Cu	10.295551	8.712247	0.000000
Cu	9.008614	6.483207	0.000000
Cu	16.730239	2.025127	0.000000
Cu	-2.573824	4.254167	0.000000
Cu	10.295551	4.254167	0.000000
Cu	14.156364	2.025127	0.000000
Cu	15.443301	4.254167	0.000000
Cu	14.156364	6.483207	0.000000
H	13.546692	4.914954	7.039118
H	12.860555	6.569290	7.109215
H	12.269183	5.314253	8.241014
H	11.076712	4.258437	6.465704
C	12.625404	5.499245	7.214825

Transition state of $*CH_3OH \rightarrow *CH_3O + *H$ on Cu/ZnO E= -430.79

O	11.735269	4.991886	6.221295
O	8.601952	0.484005	6.598915
O	5.160166	6.446134	6.751906
O	6.885656	3.456537	6.805943
O	1.724872	6.436317	6.870178
O	5.159389	0.485244	6.834499
O	3.440710	3.463759	6.802449
O	10.089098	3.246294	6.391481
O	11.836893	0.323865	6.232604
O	8.258705	6.174093	5.858323
Zn	3.430419	7.392317	6.621651
Zn	0.012010	7.426368	6.749237
Zn	1.743275	4.461753	6.673922
Zn	5.166441	4.442432	6.559779
Zn	6.849205	1.490283	6.591272
Zn	3.475735	1.499957	6.581997
Zn	8.493214	4.314018	6.426558
Zn	6.777732	7.356260	6.422377
Zn	10.293349	1.360529	6.536852
Cu	-0.012485	2.790928	4.238230
Cu	6.386142	4.975321	4.138832
Cu	5.106890	2.754446	4.175622
Cu	2.479436	7.173766	4.149445

Cu	1.293039	0.548957	4.243368
Cu	7.642664	2.748369	4.152654
Cu	1.237062	4.958351	4.189299
Cu	3.795179	0.522687	4.214151
Cu	-1.294437	5.008397	4.233877
Cu	8.871274	0.480755	4.296731
Cu	-0.065128	7.198001	4.170247
Cu	6.326763	0.506419	4.157771
Cu	3.813774	4.971140	4.158517
Cu	-2.572692	7.223252	4.234991
Cu	5.067815	7.176112	4.184779
Cu	2.526230	2.756743	4.306513
Cu	-3.860813	7.969264	2.101587
Cu	0.000000	1.282144	2.101587
Cu	2.573875	1.282144	2.101587
Cu	5.147750	1.282144	2.101587
Cu	7.721625	1.282144	2.101587
Cu	-1.286937	3.511184	2.101587
Cu	-2.573875	5.740224	2.101587
Cu	1.286938	3.511184	2.101587
Cu	6.434688	3.511184	2.101587
Cu	3.860812	3.511184	2.101587
Cu	-0.000000	5.740224	2.101587
Cu	2.573875	5.740224	2.101587
Cu	5.147750	5.740224	2.101587
Cu	-1.286937	7.969264	2.101587
Cu	1.286937	7.969264	2.101587
Cu	3.860812	7.969264	2.101587
Cu	2.573926	4.254167	0.000000
Cu	2.573926	8.712247	0.000000
Cu	6.434739	6.483207	0.000000
Cu	5.147801	8.712247	0.000000
Cu	1.286989	6.483207	0.000000
Cu	-2.573824	8.712247	0.000000
Cu	1.286989	2.025127	0.000000
Cu	9.008614	2.025127	0.000000
Cu	0.000051	8.712247	0.000000
Cu	-1.286886	6.483207	0.000000
Cu	6.434739	2.025127	0.000000
Cu	7.721676	4.254167	0.000000
Cu	0.000051	4.254167	0.000000
Cu	3.860864	2.025127	0.000000
Cu	5.147801	4.254167	0.000000
Cu	3.860864	6.483207	0.000000
Cu	10.240499	2.826089	4.227432
Cu	16.762009	5.012526	4.169158
Cu	15.460882	2.801321	4.189664
Cu	12.932382	7.318272	4.152826
Cu	11.602766	0.529241	4.245165
Cu	18.029406	2.792933	4.177037
Cu	11.683489	5.093249	4.207802
Cu	14.211227	0.593193	4.177568
Cu	9.026785	5.056903	4.087210
Cu	19.326533	0.585323	4.180834
Cu	10.361656	7.317475	4.167995
Cu	16.759742	0.590030	4.201197
Cu	14.240008	5.025545	4.143128
Cu	7.774811	7.222270	4.106649
Cu	15.459906	7.259529	4.165709
Cu	12.912174	2.791339	4.126030
Cu	6.434688	7.969264	2.101587
Cu	10.295500	1.282144	2.101587
Cu	12.869375	1.282144	2.101587
Cu	15.443250	1.282144	2.101587
Cu	18.017125	1.282144	2.101587
Cu	9.008563	3.511184	2.101587
Cu	7.721625	5.740224	2.101587
Cu	11.582438	3.511184	2.101587
Cu	16.730188	3.511184	2.101587
Cu	14.156313	3.511184	2.101587
Cu	10.295500	5.740224	2.101587
Cu	12.869375	5.740224	2.101587
Cu	15.443250	5.740224	2.101587
Cu	9.008563	7.969264	2.101587
Cu	11.582438	7.969264	2.101587
Cu	14.156313	7.969264	2.101587
Cu	12.869426	4.254167	0.000000
Cu	12.869426	8.712247	0.000000
Cu	-3.860761	6.483207	0.000000
Cu	-5.147699	8.712247	0.000000
Cu	11.582489	6.483207	0.000000
Cu	7.721676	8.712247	0.000000
Cu	11.582489	2.025127	0.000000
Cu	-1.286886	2.025127	0.000000
Cu	10.295551	8.712247	0.000000
Cu	9.008614	6.483207	0.000000
Cu	16.730239	2.025127	0.000000
Cu	-2.573824	4.254167	0.000000
Cu	10.295551	4.254167	0.000000
Cu	14.156364	2.025127	0.000000
Cu	15.443301	4.254167	0.000000
Cu	14.156364	6.483207	0.000000
H	13.680042	4.820474	7.002585
H	12.881611	6.413924	7.191928
H	12.356801	5.027006	8.197745
H	11.071869	4.129991	6.435383
C	12.716186	5.324555	7.198964

Final state of *CH_3OH → *CH_3O + *H on Cu/ZnO E= -431.17

O	12.958021	4.271919	5.494491
O	8.601952	0.484005	6.598915
O	5.160166	6.446134	6.751906
O	6.885656	3.456537	6.805943
O	1.724872	6.436317	6.870178
O	5.159389	0.485244	6.834499
O	3.440710	3.463759	6.802449
O	9.952779	3.324174	7.163205
O	11.775914	0.430188	6.277404
O	8.225964	6.291135	5.956098
Zn	3.430419	7.392317	6.621651
Zn	0.012010	7.426368	6.749237
Zn	1.743275	4.461753	6.673922
Zn	5.166441	4.442432	6.559779
Zn	6.849205	1.490283	6.591272
Zn	3.475735	1.499957	6.581997
Zn	8.458373	4.426622	6.475641
Zn	6.734755	7.459425	6.457244
Zn	10.230182	1.480122	6.578211
Cu	0.000487	2.791750	4.239029
Cu	6.393470	4.966821	4.140464
Cu	5.120643	2.749707	4.169546
Cu	2.496617	7.193061	4.134964
Cu	1.299048	0.554590	4.240515
Cu	7.680509	2.773030	4.187109
Cu	1.246942	4.966533	4.193807
Cu	3.802163	0.521704	4.213722
Cu	-1.290689	5.008686	4.239272
Cu	8.896130	0.507799	4.320790
Cu	-0.058291	7.200384	4.171026
Cu	6.346544	0.517815	4.148598
Cu	3.815437	4.971365	4.156050
Cu	-2.563733	7.219668	4.244647
Cu	5.077478	7.167756	4.189758
Cu	2.537369	2.757768	4.303346
Cu	-3.860812	7.969264	2.101587
Cu	-0.000000	1.282144	2.101587
Cu	2.573875	1.282144	2.101587
Cu	5.147750	1.282144	2.101587
Cu	7.721625	1.282144	2.101587
Cu	-1.286937	3.511184	2.101587
Cu	-2.573875	5.740224	2.101587
Cu	1.286938	3.511184	2.101587
Cu	6.434688	3.511184	2.101587
Cu	3.860812	3.511184	2.101587
Cu	0.000000	5.740224	2.101587
Cu	2.573875	5.740224	2.101587
Cu	5.147750	5.740224	2.101587
Cu	-1.286937	7.969264	2.101587
Cu	1.286937	7.969264	2.101587
Cu	3.860813	7.969264	2.101587
Cu	2.573926	4.254167	0.000000
Cu	2.573926	8.712247	0.000000
Cu	6.434739	6.483207	0.000000
Cu	5.147801	8.712247	0.000000
Cu	1.286989	6.483207	0.000000
Cu	-2.573824	8.712247	0.000000
Cu	1.286989	2.025127	0.000000
Cu	9.008614	2.025127	0.000000
Cu	0.000051	8.712247	0.000000
Cu	-1.286886	6.483207	0.000000
Cu	6.434739	2.025127	0.000000
Cu	7.721676	4.254167	0.000000
Cu	0.000051	4.254167	0.000000
Cu	3.860864	2.025127	0.000000
Cu	5.147801	4.254167	0.000000
Cu	3.860864	6.483207	0.000000
Cu	10.229793	2.764086	4.127452
Cu	16.770907	5.011776	4.157838
Cu	15.506294	2.764872	4.151658
Cu	12.898965	7.305469	4.153039
Cu	11.577656	0.511554	4.274257
Cu	18.043453	2.784285	4.173075
Cu	11.543820	5.053448	4.202295
Cu	14.225293	0.545317	4.153095
Cu	9.045410	4.989485	4.103497
Cu	19.332336	0.574923	4.183185
Cu	10.355564	7.267095	4.176914
Cu	16.752534	0.565150	4.201334
Cu	14.270550	5.072978	4.171513
Cu	7.781477	7.182764	4.145116
Cu	15.471667	7.278764	4.162299
Cu	12.894514	2.696621	4.181639
Cu	6.434687	7.969264	2.101587
Cu	10.295500	1.282144	2.101587
Cu	12.869375	1.282144	2.101587
Cu	15.443250	1.282144	2.101587
Cu	18.017125	1.282144	2.101587
Cu	9.008562	3.511184	2.101587
Cu	7.721625	5.740224	2.101587
Cu	11.582438	3.511184	2.101587
Cu	16.730187	3.511184	2.101587
Cu	14.156313	3.511184	2.101587
Cu	10.295500	5.740224	2.101587
Cu	12.869375	5.740224	2.101587
Cu	15.443250	5.740224	2.101587
Cu	9.008562	7.969264	2.101587
Cu	11.582438	7.969264	2.101587
Cu	14.156312	7.969264	2.101587
Cu	12.869426	4.254167	0.000000
Cu	12.869426	8.712247	0.000000
Cu	-3.860761	6.483207	0.000000
Cu	-5.147699	8.712247	0.000000
Cu	11.582489	6.483207	0.000000
Cu	7.721676	8.712247	0.000000
Cu	11.582489	2.025127	0.000000
Cu	-1.286886	2.025127	0.000000
Cu	10.295551	8.712247	0.000000
Cu	9.008614	6.483207	0.000000
Cu	16.730239	2.025127	0.000000
Cu	-2.573824	4.254167	0.000000
Cu	10.295551	4.254167	0.000000
Cu	14.156364	2.025127	0.000000
Cu	15.443301	4.254167	0.000000
Cu	14.156364	6.483207	0.000000
H	14.159009	4.641031	7.170361
H	12.439553	5.068438	7.357010
H	12.956508	3.333584	7.360581
H	10.791735	3.820353	7.149114
C	13.133552	4.328343	6.918480

Initial state of *OH → *O + *H on Cu/ZnO E= -411.00

O	12.911026	4.260451	5.400163
O	8.601952	0.484005	6.598915
O	5.160166	6.446134	6.751906
O	6.885656	3.456537	6.805943
O	1.724872	6.436317	6.870178
O	5.159389	0.485244	6.834499
O	3.440710	3.463759	6.802449
O	10.088268	3.261916	6.205476
O	11.807451	0.329294	6.217935
O	8.227351	6.160774	5.854171
Zn	3.430419	7.392317	6.621651
Zn	0.012010	7.426368	6.749237
Zn	1.743275	4.461753	6.673922
Zn	5.166441	4.442432	6.559779
Zn	6.849205	1.490283	6.591272
Zn	3.475735	1.499957	6.581997
Zn	8.517638	4.299177	6.429374
Zn	6.769135	7.364771	6.416032
Zn	10.276965	1.399984	6.513480
Cu	-0.004913	2.796459	4.240718
Cu	6.382384	4.972918	4.142041
Cu	5.094162	2.749049	4.178863
Cu	2.476908	7.172807	4.148073
Cu	1.301916	0.556631	4.241844
Cu	7.617572	2.744559	4.156290
Cu	1.238022	4.964957	4.191409
Cu	3.794145	0.522683	4.212204
Cu	-1.289810	5.009159	4.237043
Cu	8.866372	0.476371	4.292346
Cu	-0.066840	7.200017	4.171387
Cu	6.330061	0.504386	4.157302
Cu	3.802001	4.970012	4.161325
Cu	-2.563069	7.222546	4.239466
Cu	5.064114	7.167978	4.185820
Cu	2.525268	2.757403	4.305039
Cu	-3.860813	7.969264	2.101587
Cu	0.000000	1.282144	2.101587
Cu	2.573875	1.282144	2.101587
Cu	5.147750	1.282144	2.101587
Cu	7.721625	1.282144	2.101587
Cu	-1.286937	3.511184	2.101587
Cu	-2.573875	5.740224	2.101587
Cu	1.286938	3.511184	2.101587
Cu	6.434688	3.511184	2.101587
Cu	3.860812	3.511184	2.101587
Cu	0.000000	5.740224	2.101587
Cu	2.573875	5.740224	2.101587
Cu	5.147750	5.740224	2.101587
Cu	-1.286938	7.969264	2.101587
Cu	1.286937	7.969264	2.101587
Cu	3.860812	7.969264	2.101587
Cu	2.573926	4.254168	0.000000
Cu	2.573926	8.712247	0.000000
Cu	6.434739	6.483207	0.000000
Cu	5.147801	8.712247	0.000000
Cu	1.286989	6.483207	0.000000
Cu	-2.573824	8.712247	0.000000
Cu	1.286989	2.025127	0.000000
Cu	9.008614	2.025127	0.000000
Cu	0.000051	8.712247	0.000000
Cu	-1.286886	6.483207	0.000000
Cu	6.434739	2.025127	0.000000
Cu	7.721676	4.254167	0.000000
Cu	0.000051	4.254167	0.000000
Cu	3.860864	2.025127	0.000000
Cu	5.147801	4.254167	0.000000
Cu	3.860864	6.483207	0.000000
Cu	10.214059	2.834971	4.228111
Cu	16.779477	5.017289	4.159786
Cu	15.510559	2.772757	4.158003
Cu	12.904584	7.318472	4.156122
Cu	11.586993	0.490158	4.234500
Cu	18.046228	2.790678	4.180859
Cu	11.542776	5.104472	4.187024
Cu	14.230333	0.557361	4.156931
Cu	9.007410	5.068201	4.087995
Cu	19.337239	0.579595	4.176519
Cu	10.358632	7.305147	4.173429
Cu	16.759583	0.580188	4.208818
Cu	14.300706	5.094168	4.216633
Cu	7.760816	7.220420	4.103524
Cu	15.477695	7.288088	4.160554
Cu	12.895291	2.680838	4.166668
Cu	6.434688	7.969264	2.101587
Cu	10.295500	1.282144	2.101587
Cu	12.869375	1.282144	2.101587
Cu	15.443250	1.282144	2.101587
Cu	18.017125	1.282144	2.101587
Cu	9.008563	3.511184	2.101587
Cu	7.721625	5.740224	2.101587
Cu	11.582438	3.511184	2.101587
Cu	16.730187	3.511184	2.101587
Cu	14.156313	3.511184	2.101587
Cu	10.295500	5.740224	2.101587
Cu	12.869375	5.740224	2.101587
Cu	15.443250	5.740224	2.101587
Cu	9.008563	7.969264	2.101587
Cu	11.582438	7.969264	2.101587
Cu	14.156312	7.969264	2.101587
Cu	12.869426	4.254167	0.000000
Cu	12.869426	8.712247	0.000000
Cu	-3.860761	6.483207	0.000000
Cu	-5.147699	8.712247	0.000000
Cu	11.582489	6.483207	0.000000
Cu	7.721676	8.712247	0.000000
Cu	11.582489	2.025127	0.000000
Cu	-1.286886	2.025127	0.000000
Cu	10.295551	8.712247	0.000000
Cu	9.008614	6.483207	0.000000
Cu	16.730239	2.025127	0.000000
Cu	-2.573824	4.254167	0.000000
Cu	10.295551	4.254167	0.000000
Cu	14.156364	2.025127	0.000000
Cu	15.443301	4.254167	0.000000
Cu	14.156364	6.483207	0.000000
H	12.713853	4.203515	6.352601

Transition state of *OH → *O + *H on Cu/ZnO E= -410.84

O	12.344121	4.043319	5.501000
O	8.601952	0.484005	6.598915
O	5.160166	6.446134	6.751906
O	6.885656	3.456537	6.805943
O	1.724872	6.436317	6.870178

O	5.159389	0.485244	6.834499
O	3.440710	3.463759	6.802449
O	10.215609	3.267727	6.439415
O	11.826525	0.290836	6.213386
O	8.251763	6.128854	5.858917
Zn	3.430419	7.392317	6.621651
Zn	0.012010	7.426368	6.749237
Zn	1.743275	4.461753	6.673922
Zn	5.166441	4.442432	6.559779
Zn	6.849205	1.490283	6.591272
Zn	3.475735	1.499957	6.581997
Zn	8.555933	4.265362	6.453573
Zn	6.795037	7.327935	6.428375
Zn	10.303831	1.356138	6.550087
Cu	-0.019616	2.786407	4.238523
Cu	6.374633	4.966317	4.145064
Cu	5.099780	2.744159	4.175917
Cu	2.471362	7.166855	4.149799
Cu	1.287061	0.537148	4.239828
Cu	7.626295	2.744491	4.159109
Cu	1.223090	4.952867	4.194712
Cu	3.785295	0.516566	4.213839
Cu	-1.317847	5.007598	4.234013
Cu	8.870067	0.473299	4.304925
Cu	-0.074380	7.195687	4.171090
Cu	6.324509	0.502722	4.157793
Cu	3.795865	4.965905	4.160318
Cu	-2.587855	7.210340	4.236962
Cu	5.060564	7.159866	4.192538
Cu	2.516388	2.748761	4.311596
Cu	-3.860813	7.969264	2.101587
Cu	0.000000	1.282144	2.101587
Cu	2.573875	1.282144	2.101587
Cu	5.147750	1.282144	2.101587
Cu	7.721625	1.282144	2.101587
Cu	-1.286938	3.511184	2.101587
Cu	-2.573875	5.740224	2.101587
Cu	1.286938	3.511184	2.101587
Cu	6.434688	3.511184	2.101587
Cu	3.860812	3.511184	2.101587
Cu	0.000000	5.740224	2.101587
Cu	2.573875	5.740224	2.101587
Cu	5.147750	5.740224	2.101587
Cu	-1.286938	7.969264	2.101587
Cu	1.286937	7.969264	2.101587
Cu	3.860813	7.969264	2.101587
Cu	2.573926	4.254167	0.000000
Cu	2.573926	8.712247	0.000000
Cu	6.434739	6.483207	0.000000
Cu	5.147801	8.712247	0.000000
Cu	1.286989	6.483207	0.000000
Cu	-2.573824	8.712247	0.000000
Cu	1.286989	2.025127	0.000000
Cu	9.008614	2.025127	0.000000
Cu	0.000051	8.712247	0.000000
Cu	-1.286886	6.483207	0.000000
Cu	6.434739	2.025127	0.000000
Cu	7.721676	4.254167	0.000000
Cu	0.000051	4.254167	0.000000
Cu	3.860864	2.025127	0.000000
Cu	5.147801	4.254167	0.000000
Cu	3.860864	6.483207	0.000000
Cu	10.190207	2.791045	4.195724
Cu	16.718457	5.006378	4.159123
Cu	15.470939	2.783665	4.165662
Cu	12.897268	7.311827	4.166002
Cu	11.578496	0.485448	4.237527
Cu	18.028089	2.792575	4.175351
Cu	11.548632	5.142257	4.152700
Cu	14.211198	0.573095	4.155809
Cu	8.998923	5.049385	4.101832
Cu	19.323743	0.575700	4.182731
Cu	10.339079	7.316275	4.165379
Cu	16.751183	0.585855	4.207317
Cu	14.164137	5.019766	4.244327
Cu	7.766526	7.216984	4.104138
Cu	15.433079	7.249734	4.161301
Cu	12.891031	2.721089	4.212645
Cu	6.434687	7.969264	2.101587
Cu	10.295500	1.282144	2.101587
Cu	12.869375	1.282144	2.101587
Cu	15.443250	1.282144	2.101587
Cu	18.017125	1.282144	2.101587
Cu	9.008562	3.511184	2.101587
Cu	7.721625	5.740224	2.101587
Cu	11.582438	3.511184	2.101587
Cu	16.730187	3.511184	2.101587
Cu	14.156313	3.511184	2.101587
Cu	10.295500	5.740224	2.101587
Cu	12.869375	5.740224	2.101587
Cu	15.443250	5.740224	2.101587
Cu	9.008562	7.969264	2.101587
Cu	11.582438	7.969264	2.101587
Cu	14.156312	7.969264	2.101587
Cu	12.869426	4.254167	0.000000
Cu	12.869426	8.712247	0.000000
Cu	-3.860761	6.483207	0.000000
Cu	-5.147699	8.712247	0.000000
Cu	11.582489	6.483207	0.000000
Cu	7.721676	8.712247	0.000000
Cu	11.582489	2.025127	0.000000
Cu	-1.286886	2.025127	0.000000
Cu	10.295551	8.712247	0.000000
Cu	9.008614	6.483207	0.000000
Cu	16.730239	2.025127	0.000000
Cu	-2.573824	4.254167	0.000000
Cu	10.295551	4.254167	0.000000
Cu	14.156364	2.025127	0.000000
Cu	15.443301	4.254167	0.000000
Cu	14.156364	6.483207	0.000000
H	11.316200	3.716097	6.099526

Final state of *OH → *O + *H on Cu/ZnO   E= -411.28

O	12.823703	4.235356	5.330345
O	8.601952	0.484005	6.598915
O	5.160166	6.446134	6.751906
O	6.885656	3.456537	6.805943
O	1.724872	6.436317	6.870178
O	5.159389	0.485244	6.834499
O	3.440710	3.463759	6.802449
O	10.112331	3.332119	6.984891
O	11.793809	0.360364	6.248031
O	8.228420	6.213086	5.907879
Zn	3.430419	7.392317	6.621651
Zn	0.012010	7.426368	6.749237
Zn	1.743275	4.461753	6.673922
Zn	5.166441	4.442432	6.559779
Zn	6.849205	1.490283	6.591272
Zn	3.475735	1.499957	6.581997
Zn	8.505829	4.361568	6.490263
Zn	6.765554	7.395970	6.450251
Zn	10.271271	1.427781	6.580935
Cu	-0.010400	2.783868	4.236166
Cu	6.384782	4.957772	4.141204
Cu	5.112594	2.738848	4.167633
Cu	2.482719	7.178535	4.143702
Cu	1.287016	0.540925	4.238699
Cu	7.665949	2.756698	4.176693
Cu	1.229485	4.956774	4.193972
Cu	3.791585	0.516021	4.213963
Cu	-1.307604	5.001170	4.237133
Cu	8.894161	0.492636	4.320961
Cu	-0.069458	7.192486	4.171649
Cu	6.337017	0.505424	4.152189
Cu	3.803870	4.961551	4.157942
Cu	-2.575329	7.212377	4.241047
Cu	5.063447	7.158438	4.191967
Cu	2.525008	2.748784	4.306172
Cu	-3.860813	7.969264	2.101587
Cu	-0.000000	1.282144	2.101587
Cu	2.573875	1.282144	2.101587
Cu	5.147750	1.282144	2.101587
Cu	7.721625	1.282144	2.101587
Cu	-1.286938	3.511184	2.101587
Cu	-2.573875	5.740224	2.101587
Cu	1.286938	3.511184	2.101587
Cu	6.434688	3.511184	2.101587
Cu	3.860812	3.511184	2.101587
Cu	0.000000	5.740224	2.101587
Cu	2.573875	5.740224	2.101587
Cu	5.147750	5.740224	2.101587
Cu	-1.286938	7.969264	2.101587
Cu	1.286937	7.969264	2.101587
Cu	3.860813	7.969264	2.101587
Cu	2.573926	4.254167	0.000000
Cu	2.573926	8.712247	0.000000
Cu	6.434739	6.483207	0.000000
Cu	5.147801	8.712247	0.000000
Cu	1.286989	6.483207	0.000000
Cu	-2.573824	8.712247	0.000000
Cu	1.286989	2.025127	0.000000
Cu	9.008614	2.025127	0.000000
Cu	0.000051	8.712247	0.000000
Cu	-1.286886	6.483207	0.000000
Cu	6.434739	2.025127	0.000000
Cu	7.721676	4.254167	0.000000
Cu	0.000051	4.254167	0.000000
Cu	3.860864	2.025127	0.000000
Cu	5.147801	4.254167	0.000000
Cu	3.860864	6.483207	0.000000
Cu	10.210864	2.754838	4.126594
Cu	16.742267	5.005193	4.150699
Cu	15.495566	2.762995	4.160531
Cu	12.892191	7.302699	4.157594
Cu	11.581839	0.494854	4.257211
Cu	18.030226	2.780925	4.171426
Cu	11.530168	5.053680	4.200810
Cu	14.207415	0.544195	4.150031
Cu	9.026722	4.997201	4.106312
Cu	19.323164	0.564923	4.180486
Cu	10.344589	7.268655	4.162061
Cu	16.743530	0.561766	4.199267
Cu	14.216151	5.042725	4.271369
Cu	7.772011	7.183081	4.126707
Cu	15.459666	7.253404	4.154324
Cu	12.879083	2.694975	4.205594
Cu	6.434687	7.969264	2.101587
Cu	10.295500	1.282144	2.101587
Cu	12.869375	1.282144	2.101587
Cu	15.443250	1.282144	2.101587
Cu	18.017125	1.282144	2.101587
Cu	9.008562	3.511184	2.101587
Cu	7.721625	5.740224	2.101587
Cu	11.582438	3.511184	2.101587
Cu	16.730187	3.511184	2.101587
Cu	14.156313	3.511184	2.101587
Cu	10.295500	5.740224	2.101587
Cu	12.869375	5.740224	2.101587
Cu	15.443250	5.740224	2.101587
Cu	9.008562	7.969264	2.101587
Cu	11.582438	7.969264	2.101587
Cu	14.156312	7.969264	2.101587
Cu	12.869426	4.254167	0.000000
Cu	12.869426	8.712247	0.000000
Cu	-3.860761	6.483207	0.000000
Cu	-5.147699	8.712247	0.000000
Cu	11.582489	6.483207	0.000000
Cu	7.721676	8.712247	0.000000
Cu	11.582489	2.025127	0.000000
Cu	-1.286886	2.025127	0.000000
Cu	10.295551	8.712247	0.000000
Cu	9.008614	6.483207	0.000000
Cu	16.730239	2.025127	0.000000
Cu	-2.573824	4.254167	0.000000
Cu	10.295551	4.254167	0.000000
Cu	14.156364	2.025127	0.000000
Cu	15.443301	4.254167	0.000000
Cu	14.156364	6.483207	0.000000
H	10.944019	3.788305	6.726025

S11. References

(1) Grimme, S.; Ehrlich, S.; Goerigk, L. Effect of the Damping Function in Dispersion Corrected Density Functional Theory. *J. Comput. Chem.* **2011**, *32*, 1456−1465.
